# Peptide‐functionalized double network hydrogel with compressible shape memory effect for intervertebral disc regeneration

**DOI:** 10.1002/btm2.10447

**Published:** 2022-11-17

**Authors:** Chia‐Yu Ho, Chen‐Chie Wang, Tsung‐Chiao Wu, Chen‐Hsiang Kuan, Yu‐Chung Liu, Tzu‐Wei Wang

**Affiliations:** ^1^ Department of Materials Science and Engineering National Tsing Hua University Hsinchu Taiwan; ^2^ Department of Orthopedic Surgery Taipei Tzu Chi Hospital, Buddhist Tzu Chi Medical Foundation New Taipei City Taiwan; ^3^ Department of Orthopedics, School of Medicine Tzu Chi University Hualien Taiwan; ^4^ Division of Plastic Surgery, Department of Surgery National Taiwan University Hospital Taipei Taiwan; ^5^ Graduate Institute of Clinical Medicine, College of Medicine National Taiwan University Taipei Taiwan; ^6^ Research Center for Developmental Biology and Regenerative Medicine National Taiwan University Taipei Taiwan

**Keywords:** biomimicry, double network hydrogel, intervertebral disc, mesenchymal stem cell, peptide functionalization, shape memory effect

## Abstract

As a prominent approach to treat intervertebral disc (IVD) degeneration, disc transplantation still falls short to fully reconstruct and restore the function of native IVD. Here, we introduce an IVD scaffold consists of a cellulose‐alginate double network hydrogel‐based annulus fibrosus (AF) and a cellulose hydrogel‐based nucleus pulposus (NP). This scaffold mimics native IVD structure and controls the delivery of Growth Differentiation Factor‐5 (GDF‐5), which induces differentiation of endogenous mesenchymal stem cells (MSCs). In addition, this IVD scaffold has modifications on MSC homing peptide and RGD peptide which facilitate the recruitment of MSCs to injured area and enhances their cell adhesion property. The benefits of this double network hydrogel are high compressibility, shape memory effect, and mechanical strength comparable to native IVD. In vivo animal study demonstrates successful reconstruction of injured IVD including both AF and NP. These findings suggest that this double network hydrogel can serve as a promising approach to IVD regeneration with other potential biomedical applications.

## 1 INTRODUCTION

Degenerative disc disease (DDD) entails the breakdown of one or more intervertebral discs (IVD). Common symptoms include disc bulging, herniation, and the formation of osteophyte (bone spurs). These conditions often interfere with nervous structures and cause pain in back or neck which can eventually lead to peripheral neuropathy.^1^, ^2^ Causes for disc degeneration are numerous, such as aging, acute overloading, or long‐term stress. These factors often result in progressive decline in disc nutrient supply and changes in extracellular matrix (ECM) composition, thereby weakening tissue strength and altering cellular metabolism.^3^ Unfortunately, the self‐repair ability of IVDs is poor, and the degeneration process is often irreversible.^4^

Surgical interventions to remove the injured/degenerated IVD and replace with artificial device are often applied when the disc can no more maintain cellular activity and biochemical environment. Although total disc replacement (TDR) has been regarded as a promising approach to reduce pain and reverse degeneration, the implanted scaffolds still have several drawbacks. First, compared with human cortical and cancellous bone, metallic TDR possesses extremely high elastic modulus, which can lead to the occurrence of stress‐shielding effect between implantation site and intervertebral body.^5^, ^6^ Second, polymer‐based scaffolds often fail to maintain integrity under constant compressive loading.^7^ Failure to recover to full disc height runs the risk of material leakage. Third, low wear resistance of polymer increases the risk of osteolysis over long‐term implantation.^8^, ^9^ Moreover, the invasive surgical procedure during implantation itself also raises a challenge. Insertion of artificial discs often requires surgical approaches both anteriorly and posteriorly to the intervertebral space, posing high risk of complications and resulting in prolonged recovery time in patients.^10^ Facing these issues, the aim of this research is to overcome the mechanical and biochemical limitations of conventional IVD implants and simplify the surgery to minimize the risk of morbidity.

Double network (DN) hydrogel has been introduced to strengthen and toughen the polymeric scaffold. It consists of two different types of network structure: one chemically crosslinked network and one physically crosslinked network.^11^ In the former structure, polymer chains form a rigid bulk for DN hydrogels and maintain the shape. The later structure fills in the rigid network and absorbs excess energy.^12^ Admittedly, physically crosslinked network has a less stable structure, unlike its chemically crosslinked counterpart. However, hydrogel formed by non‐covalent bonds can usually undergo reversible process, which the bonding can immediately recover after breaking.^13^ One major challenge that current polymer‐based hydrogel faces is to fully replicate the biomechanical properties required to support multiaxial spinal loads. The elastic modulus of native IVD is about 0.5 to 1 MPa. Through the design of DN hydrogel, the polymer scaffold can enhance its mechanical strength to meet the needs of engineered discs.

Shape memory polymers (SMPs) are smart materials capable of recovering to a pre‐determined shape in response to external stimuli.^14^, ^15^ Such property could be applied to simplify the implantation procedure as minimally invasive surgery. Besides, SMP scaffolds exhibit a self‐fitting capacity from the force generated during shape recovery process.^16^ The capacity ensures the implanted hydrogel to tightly fit in the original space of degenerative disc. The rigid contact can prevent material sliding by forming a constraint force between the scaffold and adjacent vertebrae.

Mesenchymal stem cells (MSCs) play an important role in tissue regeneration from their abilities to sustain long‐term self‐renewal and differentiate into other cell types.^17^ Critically, MSCs engage by migration to the wound when injured.^18^ By mimicking the configuration of bone marrow homing peptide (BMHP), a short peptide sequence SKPPGTSS (SKP) has been reported to facilitate MSC homing.^19^, ^20^ With the engraftment of SKP peptide, the hydrogel serves as a platform for MSCs homing. Meanwhile, for supporting in‐growth and adhesion of endogenous stem cells, cell adhesion peptide sequence GRGDSP (RGD) is designed and immobilized on polymer network to augment cell/matrix interactions.^21^, ^22^

Various growth factors have been proposed in vitro to stimulate ECM synthesis by IVD cells, including transforming growth factor‐β (TGF‐β), insulin‐like growth factor‐1 (IGF‐1), and bone morphogenetic protein‐2 (BMP‐2).^23^, ^24^ However, the angiogenic potential of the growth factors (TGF‐β or IGF‐1) may adversely promote blood vessel ingrowth, which is undesirable in degenerative disc treatment.^25^ On the other hand, Growth Differentiation Factor‐5 (GDF‐5) receptors are non‐angiogenic, and *GDF5* gene was shown to be a susceptiblity gene for disc degeneration, suggesting that GDF‐5 can be a promising candidate to stimulate ECM.^26^ Also, GDF‐5 was reported to enhance the proliferation of both AF and NP cells, and to down‐regulate the metalloproteinase (MMP) expression by inhibiting ECM catabolism.^27^ However, current trends of delivery of GDF‐5 via single injection may breakdown the disc and thus compromise the long‐term outcome.

**FIGURE 1:**
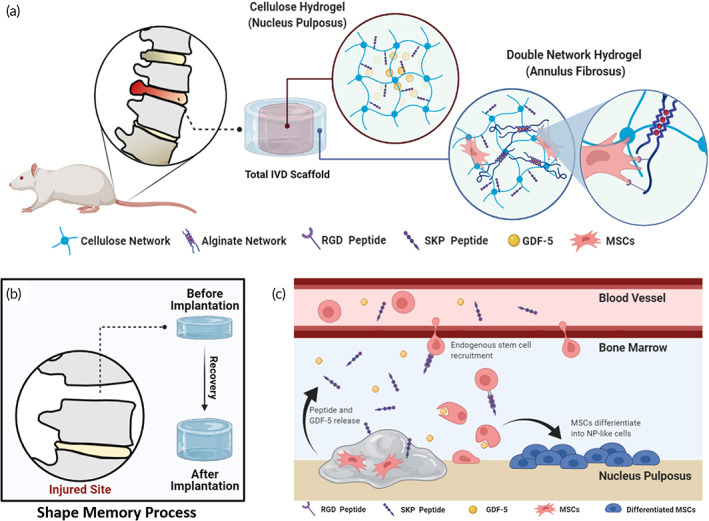
Illustration schemes and design strategies of this study. (a) Formation of peptide functionalized double network hydrogel with IVD‐mimicking structure and loaded bioactive factor. (b) Shape recovery process of double network hydrogel in caudal disc of rat. (c) Endogenous stem cell recruitment and differentiation in peptide functionalized hydrogel

Here, we aimed to develop a tough and bioactive hydrogel to replace the degenerated discs. A highly compressible DN hydrogel was synthesized via physically crosslinked alginate network and chemically crosslinked cellulose network (Figure 1a). While DN hydrogel was made into cylinder shape, serving as the AF, the NP was formed via the injection of prime cellulose solution into the central part of the scaffold. With structural mimicry of native IVD, this scaffold can provide therapeutic effect on both NP and AF. Shape memory property was also incorporated by addition of chelating agents, and the DN hydrogel could underwent compression and recovered the disc height, indicating the feasibility of minimally invasive surgery (Figure 1b). Furthermore, MSC homing peptide (SKP peptide) and cell adhesion peptide (RGD peptide) were modified on the polymer chain of cellulose and alginate, respectively (Figure 1c). The SKP peptide can recruit endogenous MSCs toward the injured site and the RGD peptide can enhance cell survival and attachment. Meantime, the MSCs can benefit from GDF‐5, which is released from the central part of scaffold, and differentiate into NP‐like cells, contributing to ECM formation and proteoglycans synthesis. Collectively, we believe that the reported hydrogel will serve as a promising alternative for total disc replacement and delay discs degeneration in combination with other treatment modalities.

## 2 MATERIALS AND METHODS

### 2.1 Functional peptide sequences modification

Peptide‐functionalized cellulose was prepared in accordance with the method described in previous study.^36^ In brief, 0.4 g of cellulose microcrystalline powder (Alfa Aesar, USA) and sodium periodate were added to 20 ml of deionized water, protected from light. After 24 h, the reaction was stopped by adding 5 ml ethylene glycol to the solution and stirring for 1 h. The reaction mixture was purified by centrifugation with deionized water to remove excess ethylene glycol. Aldehyde‐functionalized cellulose (cellulose‐ALD) was then obtained. To prepare peptide‐functionalized cellulose, Schiff base reaction was conducted between aldehyde groups of cellulose‐ALD and amino groups on the glycine of peptide sequence. In brief, 3 mg of GGSKPPGTSS (SKP peptide; Allbio, Taiwan) was dissolved in 5 ml deionized water. The solution was carefully titrated, and the pH value was adjusted to 8 by 1 N NaOH. 50 mg of cellulose‐ALD was added to the solution, and the mixture was stirred at 40 °C for 4 h. The resulting solution was then centrifuged with deionized water for three times to remove the unreacted SKP peptide followed by lyophilization for further storage.

As for the preparation of peptide‐functionalized alginate, the method was described in previous study.^37^ Shortly, 50 mg of sodium alginate (Alfa Aesar) was dissolved in a 2‐(N‐morpholino) ethanesulfonic acid (MES) buffer at room temperature (pH = 6.5, 0.3 M NaCl). 3 mg of GRGDSP peptide sequence (RGD peptide; GeneDirex, USA) was added to the alginate solution in the presence of N‐ hydroxysuccinimide (NHS; Fluka, Switzerland) and N‐(3‐Dimethylaminopropyl)‐N′‐ethylcarbodiimide (EDC; Sigma‐Aldrich, USA). 30.1 mmol of EDC and 0.05 mmol of NHS was added to the solution. The mixture was incubated at room temperature while stirring for 6 h. The resulting solution was dialyzed (MWCO = 6000) against deionized water for 24 h to remove catalysts EDC/NHS and unreacted RGD peptides. The final solution was lyophilized and stored at −20 °C for further use. The modification of functional peptide sequences was determined by ^1^H NMR (Bruker, AVANCE‐500) and FT‐IR (Bruker, Vertex 80v).

### 2.2 Preparation and characterization of DN hydrogel and total IVD scaffold

Alkali/urea solvent system was utilized to dissolve cellulose‐SKP powder.^38^ In brief, cellulose‐SKP was added to 10 ml of alkali/urea solution (0.8 g NaOH and 0.4 g Urea in 10 ml deionized water) with vigorous stirring. After 24 h storing under −80 °C, the frozen solution was thawed at room temperature. When the solution was completely thawed, it was placed the mixture back to −80 °C again. The freeze–thaw cycle was repeated for three times, and a completely dissolved cellulose solution was obtained. Next, alginate‐RGD powder was added into previous cellulose solution. Epichlorohydrin (ECH; Alfa Aesar) was added dropwise in the solution at a volume ratio of 80 μl ml^−1^. After stirring the solution for 15 min, cellulose/alginate solution was poured into preformed mold. The gelation occurred within 4 h. To crosslink alginate network, the hydrogel was then immersed into 4% (w/v) CaCl_2_ solution for 4 h. The alginate powder was added to the cellulose solution with vigorous stirring, forming a homogenous solution. Before the immersion into CaCl_2_ solution, the hydrogel remains transparent, showing there was no phase separation in the hydrogel. The phenomenon that DN hydrogel turned from transparent to white may result from the crosslinking between Ca ion and alginate (Figure S8). For the total IVD scaffold preparation, the center part of cellulose/alginate DN hydrogel was removed by a biopsy punch, forming a hollow cylinder. Then, cellulose solution was injected into the inner part until gelation; thus, a total IVD scaffold with inner and outer parts was synthesized.

Scanning electron micrographs were taken with a high‐resolution thermal field emission scanning electron microscope (SEM; JEOL, JSM‐7610F). Both cellulose hydrogel and DN hydrogel were frozen in liquid nitrogen and snapped immediately. The frozen samples were then immersed in adequate amount of water and stored in −80 °C for 24 h. After completely freezing, the samples were lyophilized consequently. The fracture surface of the hydrogel was sputtered with gold, observed, and photographed.

Swelling ratio of the hydrogels was measured at room temperature. The hydrogel samples were incubated in 1× phosphate‐buffered saline. Both the volume and weight of each hydrogel sample were measured before and after the incubation with PBS at predetermined time points. The swelling ratio of hydrogel is defined as:

$$\text{Swelling ratio}\;\left(\mathrm{SR}\right)=\frac{\text{weight of hydrogel after immersing}}{\text{weight of hydrogel before immersing}}\times 100\%$$

### 2.3 Degradation kinetics and GDF‐5 release behavior

50 μl of DN hydrogel and cellulose hydrogel was injected into a cylinder mold and set for 4 h to form a tube‐like hydrogel. After gelation, the hydrogel was transferred into a 24‐well plate. 2 ml of PBS was added in each well and incubated with the samples at 37 °C. At predetermined time points, the supernatant was completely removed and replaced with fresh PBS. Hydrogel samples were collected and stored in −80 °C for further lyophilization. The dry weight was measured at different time points.

Recombinant GDF‐5 (Abcam, USA) was dissolved in 1% bovine serum albumin (BSA) solution. To determine the release profile, 10 μl of GDF‐5 (100 μg ml^−1^) was added in 400 μl of cellulose solution with the presence of ECH. In order to simulate the release of GDF‐5 from total IVD scaffold, an in vitro release model was designed. In brief, the GDF‐5 loaded cellulose hydrogel was placed in the center of 4 ml sample bottle. Appropriate amount of cellulose/alginate solution with homogenous ECH was injected to surround the original cylinder gel. Each time the surrounding solution underwent gelation, 2 ml of release buffer (1× PBS with 1% BSA) was added carefully on the top of hydrogel. Cellulose hydrogels without the surroundings were also prepared for comparison. At predetermined time points, the supernatant was partially removed by syringe (1 ml), and replenished the solution volume to 2 ml with fresh release buffer. The collected solution was stored at −20°C for further analysis. The amount of GDF‐5 in the collected release buffer was quantified using GDF‐5 ELISA kit (Boster, USA) following the manufacturer's instructions.

### 2.4 Mechanical property test

Mechanical behaviors were tested by compression test using a universal testing machine (UTM, Instron 3400). A cylindrical hydrogel (about Ø 14 mm × 9.5 mm) was prepared as described above. Then, the hydrogel was loaded on the lower plate and compressed by the upper plate at a strain rate of 1 mm min^−1^. All samples were analyzed to obtain the stress–strain curve. As for cyclic test, total IVD scaffold was tested by repeated loading to 50% strain. Then, 20 cycles of loading and unloading were done to the hydrogel with a strain rate of 3 mm min^−1^. All samples were measured at room temperature, and the data was further analyzed using Bluehill 3 software.

### 2.5 Evaluation of shape memory properties

Shape memory process was conducted with the DN hydrogel. The hydrogel was prepared without the immersion in CaCl_2_ solution since Ca^2+^ ions were utilized to fix the temporary shape in the following procedure. Initially, the hydrogel was deformed to different degrees of compression by a clamp. Then, the deformed hydrogel with the clamp were soaked in the CaCl_2_ solution for shape fixing. Finally, excess amount of ethylenediaminetetraacetic acid (EDTA) chelating agents was added to the solution, triggering the shape recovery process. The height of hydrogel in each process was measured. Three factors were calculated, including shape fixity ratio (*R*_*f*_), shape recovery ratio (*R*_*r*_), and recovery time. These factors are defined as:

$$\text{Shape fixity ratio}\left(R_{f}\right)=\frac{H_{f}}{H_{d}}\times 100\%$$

$$\text{Shape recovery ratio}\left(R_{r}\right)=\frac{H_{F}}{H_{i}}\times 100\%$$

*H*_*i*_ is the initial height of hydrogel; *H*_*d*_ is the deformed height of hydrogel; *H*_*f*_ is the fixed height of hydrogel; and *H*_*F*_ is the final height of hydrogel. The shape recovery time records the time begins from soaking to CaCl_2_ solution to fully recovery.

### 2.6 Cell culture and biocompatibility assessment

The rat mesenchymal stem cells (rMSCs) used in this study were derived from the bone marrow of adult Fisher 344 rats. The culture medium was Dulbecco's Modified Eagle Medium‐Low Glucose (DMEM‐LG; Invitrogen, USA) supplemented with 20% fetal bovine serum (FBS) and 1% penicilline/streptomycin (PS; Gibco, USA). Cells were maintained in a 37 °C, 5% CO_2_ environment. Culture medium was replaced every 2–3 days. rMSCs at passages 3–5 were used for subsequent experiments. Hydrogels were dialyzed (MWCO 6000–8000) against DI water for 4 h to remove excess ECH, and the samples were later placed under ultraviolet for sterilization before in vitro cell culture.

Biocompatibility assessment of both cellulose hydrogel and DN hydrogel was evaluated by Live/Dead assay (Molecular Probe, USA) and lactate dehydrogenase (LDH) assay. Prepared samples were placed in 48‐well plates, and incubated at 37 °C, 5% CO_2_ for 10 min. Hydrogel samples were co‐cultured with rMSCs (1 × 10^5^ cells/well) under 1, 3, and 7 days of incubation. At predetermined time points, live and dead cells were stained with calcein AM (C‐AM, Green) and ethidium homodimer‐1 (EthD‐1, Red) for 45 min at 37 °C, respectively. The stained cells were observed by inverted fluorescent microscope (Carl Zeiss, Axiovert 40 CFL). For LDH assay, 50 μl culture medium was drawn to a 96‐well plate, and 50 μl of CytoTox 96 Reagent (Promega, USA) was then mixed to each well with extracted solution. The microplate was incubated for 30 min in dark at room temperature. Finally, after adding 50 μl Stop Solution into each well, absorbance at 490 nm was measured by SpectraMax Plus384 Microplate Reader.

### 2.7 MSC proliferation and differentiation assessment

The IVD scaffolds with or without GDF‐5 protein incorporation were fabricated and co‐cultured with rMSCs. To determine the cell proliferation, the amount of WST‐8 was quantified using Cell Counting Kit‐8 (CCK‐8 Assay; Abcam). 50 μl of hydrogel samples were placed in 48‐well plate initially. 0.5 ml of culture medium (2.5 × 10^4^ cells/well) was carefully added to each well and changed by 50% every 2 days. After incubating for 1, 3, 7, and 14 days, 50 μl of CCK‐8 solution (1/10 of medium volume) was added to each well. The microplate was then set in the incubator for 2 h until the color changed to orange. Absorbance at 450 nm was measured using SpectraMax Plus384 microplate reader.

The impact of GDF‐5 protein on rMSCs was determined by the expression levels of marker genes of chondrocytes and NP‐like cells. Conditions with and without GDF‐5 protein were tested under incubation with MSCs for 7 and 14 days. In brief, the RNA from each sample was extracted by TRIzol reagent (Invitrogen). Isolation of extracted RNA was conducted with the addition of isopropanol, ethanol, and RNase‐free water sequentially. After obtaining RNA, reverse transcript reaction was done to transcript RNA to obtain complementary DNA (cDNA). Then, Qubit 2.0 Fluorometric Quantitation Platform with Qubit RNA BR Assay and Qubit dsDNA BR Assay Kits (Thermo Fisher Scientific) were applied to quantify the concentration of extracted RNA and cDNA before conducting qPCR experiments. The obtained cDNA was used as a template in gene quantification by PCR. The differentiation of rMSCs was monitored with these specific genes: (1) cartilage‐related gene, type II collagen (COL2), (2) cartilage‐specific proteoglycan, aggrecan, (3) chondrocyte‐related gene, SOX‐9, (4) nucleus pulposus‐related gene, keratin 19 (KRT‐19), and (5) Glyceraldehyde 3‐phosphate dehydrogenase (GAPDH) was used as the housekeeping gene to normalize RNA expressions. All the primers were synthesized by Bio‐rad (USA) and their sequences were listed in Table S1. Real‐time PCR was performed using iQ SYBR Green Supermix and detected in a MiniOpticonTM (Bio‐rad) two color Real‐time PCR Detection System. The results were calculated using the $2^{-∆∆\mathrm{CT}}$ method.

Aside from qPCR, Alcian blue (Sigma‐Aldrich) and Picrosirius red solution (Abcam) were used to stain for glycosaminoglycans (GAGs) and collagen, respectively. After co‐culturing the hydrogel with and without GDF‐5 protein for 7 days, culture medium was removed, and the samples were washed with PBS for three times. The cells were fixed with 4% formaldehyde at 4 °C for 30 min. For GAG staining, rMSCs were treated with Alcian blue solution for 15 min at room temperature and washed with tap water for 5 min subsequently. For collagen staining, rMSCs were stained in Picrosirius red solution for 1 h and further washed with two changes of acidified water. The samples were dehydrated in ethanol for three times and observed with optical microscope.

### 2.8 Cell migration test

In order to verify the recruitment of rMSCs, inserts with a diameter of 6.5 mm (24‐well plate) and 8 μm pore size were used. 200 μl of solution containing rMSCs (5 × 10^4^ cells/well) in culture medium was added in each Transwell insert and incubated for 10 min at 37 °C and 5% CO_2_ to allow the cells to attach. Hydrogel samples were then added to the bottom of 24‐well plate as well as 800 μl of culture medium. At predetermined time points (24 and 72 h), the medium in Transwell inserts were aspirated and washed with PBS twice. rMSCs were stained with DAPI and H&E stain.

For fluorescent staining, rMSCs were fixed with 4% formaldehyde at 4 °C for 30 min and treated with 0.1% Triton X‐100 for 5 min. The cellular nucleus was further stained with 4′,6‐diamidino‐2‐phenylindole (DAPI, blue) (10 μg ml^−1^) for 10 min in dark. As for H&E staining, the inserts were placed into 4% formaldehyde for 5 min at room temperature to fix the cells on the membrane. The membrane was then stained with hematoxylin solution for 5 min and washed in tap water followed by stained with 1% eosin (Gills, USA) for 1 min, following washing in tap water. After the staining process, the inside of each insert was gently swabbed using cotton swabs, taking care not to damage the membrane or touch the underside of the inserts. The Transwell inserts were placed under fluorescent microscope and optical microscope to count cell numbers of the selected visual fields (5 visual fields/insert, 10×).

Direct observation of Transwell insert membrane was also conducted with the help of SEM. Medium in the inserts was removed and gently rinsed with PBS, following with fixation in 4% formaldehyde for 30 min at 4°C. The membrane was carefully removed from the insert using a scalpel blade and mounted onto the silicon wafer for further preparation. The samples were then sequentially dehydrated with 50% ethanol for 10 min, 75% ethanol for 10 min, 95% ethanol for 10 min, and immersed in 100% ethanol for 10 min (twice). Afterwards, critical point drying (CPD) was performed, and the membrane was further sputtered with gold.

### 2.9 Cell adhesion test

Hydrogel samples were prepared, placed in a 48‐well plate, and incubated at 37 °C, 5% CO_2_ for 10 min. 20 μl of rMSC suspension solution (5 × 10^3^ cells/well) was carefully seeded on the surface of each sample. The samples were set in the incubator for 1 h, allowing rMSCs to attach to the hydrogel surface. After that, 0.5 ml of rMSC culture medium was added in each well. CellTiter 96 Aqueous One Solution Cell Proliferation Assay 1000 assays (MTS assay; Promega) was conducted at 6, 12, and 24 h. rMSCs were stained with fluorescent agents and observed via an inverted confocal microscope (Carl Zeiss, LSM 800) after 24 h incubation. The adhered cells were stained by DAPI (10 μg ml^−1^) and ACTI‐Stain 488 Phalloidin (AF488, green; Millipore, USA) (25 μl ml^−1^).

### 2.10 Animal and surgical procedures

The experimental protocols were approved by the Institutional Animal Care and Use Committee (IACUC) at Laboratory Animals Center in National Tsing Hua University, Taiwan (IACUC Protocol No. 11011H056). Sprague–Dawley Rats (male, 4–6 weeks) purchased from BioLasco Taiwan Co., Ltd were used in this study. The SD Rats were randomly treated with five different conditions (*n* = 5), details were shown in Table S2. The following surgical procedure for degenerated disc disease was performed.^39^, ^40^ After the preparation of total IVD scaffolds containing functional peptide sequences and bioactive molecules, the artificial discs were implanted into rats as caudal spine discs between third and fourth caudal vertebrae (caudal 3/4) to evaluate their long‐term performance. Rats were anesthetized using zoletil (Zoletil 50, 50 mg ml^−1^; Virbac, France) (30 mg kg^−1^), and xylazine 7.5 (mg kg^−1^) (Rompun, 20 mg ml^−1^; Bayer Korea, Korea), which were mixed together and administered intramuscularly. An initial dose of 25 mg kg^−1^ cefazoline (Standard Chem & Pharm, Taiwan) was injected intramuscularly after anesthetic injection. Native disc was removed by a rongeur, and a disc space was prepared for implant insertion in the tail. The vertebral column was later exposed allowing insertion of the IVD scaffold into the original space. The disc space was released to press‐fit the implant in place and wound closure was performed with 3–0 surgical suture. There was no animal died during the experiment period.

### 2.11 Magnetic resonance imaging (MRI) analysis

For MR scanning, rats were anesthetized with isoflurane (1.5–2%) and placed in 3.0 T MRI scanner (Philps, Ingenia 3.0 T). The rat tail was held by tape to prevent movements (Figure S9). T2‐weighted images (T2WIs) were acquired using rapid acquisition with relaxation enhancement sequence with field of view = 40 × 40 mm^2^, matrix size = 192 × 192, flip angel = 180°, repetition time/echo time (TR/TE) = 4000/68 ms, and 8 slides of 1 mm thickness. Disc heights and disc degenerative degree of each group were quantified by the high signal ratio in IVD using the Image J software.^41^ The high signal ratio is defined as:

$$\text{High signal ratio in disc}=\frac{\text{High signal area of experimental condition}}{\text{High signal area of adjacent normal disc}}$$

Pfirrmann et al. introduced a grading system for disc degeneration based on MR signal intensity including disc structure, distinction between nucleus and anulus, and disc height.^42^, ^43^ In this study, we modified this grading system in order to properly evaluate the regenerative effect of our scaffold. The grading scale was shown in Table S3.

### 2.12 Histological and immunohistochemical examination

Rats were deeply anesthetized using 5% isoflurane and sacrificed. Discs as well as adjacent vertebrae were extracted and then fixed in 4% phosphate‐buffered paraformaldehyde at 4°C for 2 days followed by washed in running tap water overnight to remove residual formalin. The samples were then sequentially decalcified in DECALCIFIER II (Leica Surgipath, USA) solution for 24 h to make the bone soft enough for the following section procedure. Decalcified IVDs and scaffolds were then rinsed with PBS and embedded in paraffin. The tissues as well as hydrogels were cut into 5 μm sections. Samples were treated with Alcian blue Picrosirus red (ABPR) or hematoxylin and eosin (H&E). The cellularity and morphology were evaluated using a grading scale shown in Table S4.^44^, ^45^

AbPr staining was conducted by treating the de‐paraffinized cryosections with Alcian blue solution for 15 min followed by rinsing in running tap water for 10 min. Then, the cryosections were incubated with Picrosirus red solution. After staining for 45 min at room temperature, the samples were rinsed in 0.5% aqueous acetic acid for 5 min. The final product was then sequentially dehydrated in 95% ethanol and 100% ethanol (twice) for 2 min and mounted on the glass slide. H&E staining samples were prepared as previously described. The stained samples were observed and recorded with optical microscope. For immunochemical analysis, fixed cells and cryosections were permeabilized with 1% Triton X‐100 for 5 min and blocked with 2% BSA for 1 h at room temperature. The samples were incubated with NP‐specific primary antibody overnight at 4°C followed by three times of washing by PBS. The primary antibody treated samples were then stained with secondary antibody dilutions for 2 h at 37°C. Counter‐staining was done using DAPI in PBS (10 μg/ml) at room temperature for 10 min. The stained samples were visualized by fluorescence microscopy. The antibody dilution factors were: primary rabbit anti‐FOXF1 antibody, 1:100 (MyBioSource, USA); primary rabbit anti‐Vimentin Alexa Fluor 594 antibody, 1:200 (Abcam); secondary goat‐anti rabbit Alexa Fluor 488 antibody, 1:500 (Abcam).

### 2.13 Biomechanical property test

Motion segments and intact native motion segments were both cleaned with surrounding tissue to result in bone‐disc‐bone motion segments after sacrificing the animals at 8 weeks. The mechanical property of the IVD implants at the moving segment, consisting of the IVD and the adjacent vertebrae, was tested using UTM at a compression rate of 0.5 mm min^−1^. The height of the sample was 12 mm and the diameter of the IVD was 10 mm.

### 2.14 Statistical analysis

Data were presented with mean ± SD. Statistical significance was determined by Student's *t* test or one‐way analysis of variance (ANOVA). N.S. indicates no significance difference, * indicates *p* ≤ 0.05, ** indicates *p* ≤ 0.01, *** indicates *p* ≤ 0.001, and **** indicates *p* ≤ 0.0001.

## 3 RESULTS

### 3.1 Evaluation of the hydrogels and total IVD scaffold

**FIGURE 2:**
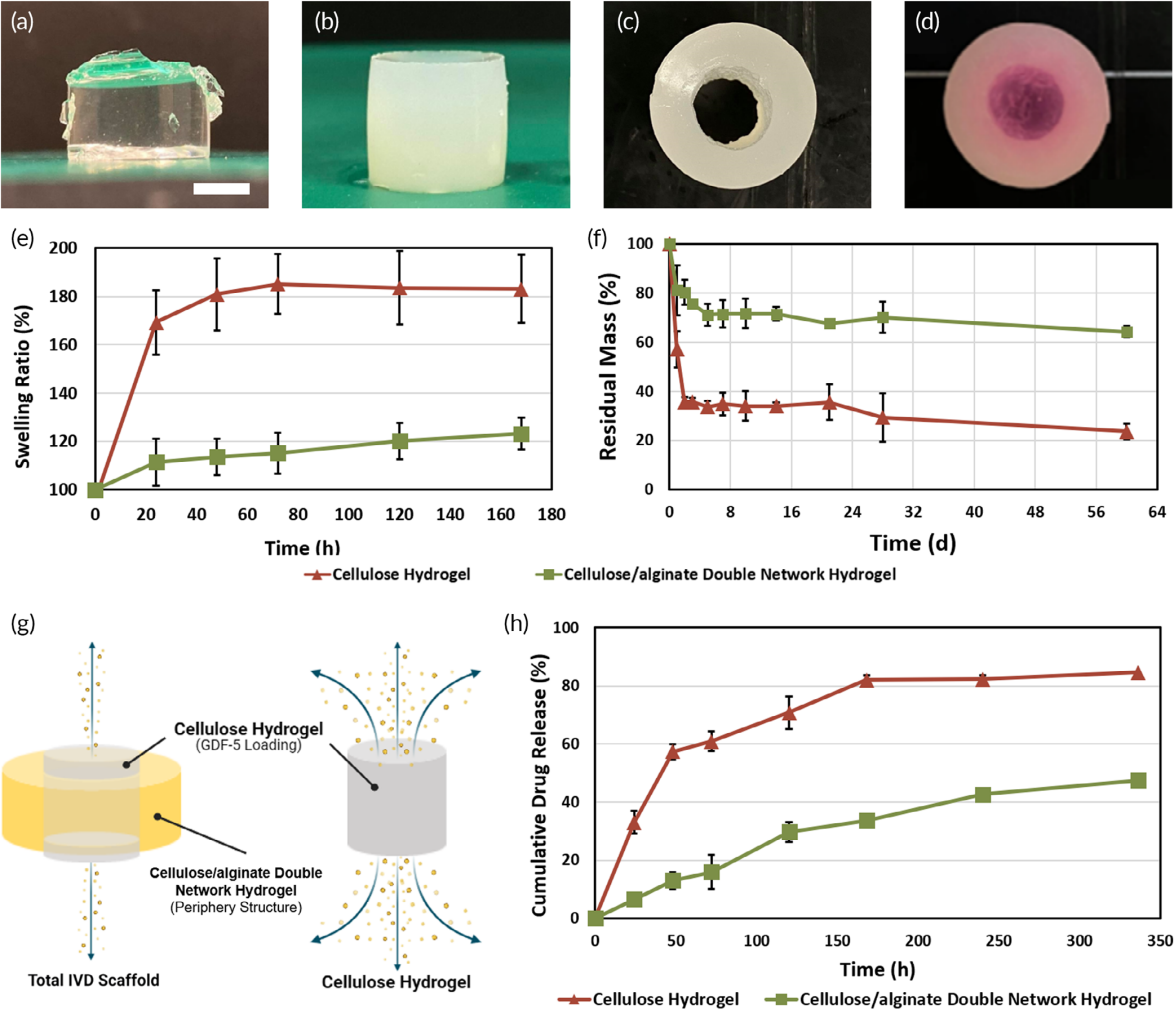
Fabrication and characterization of DN hydrogel and total IVD scaffold. (a) Cellulose hydrogel. (b) Cellulose/alginate double network hydrogel (after the immersion of CaCl_2_ solution). (c) Top view of hollow cylinder double network hydrogel, with central part removed. (d) Total IVD scaffold, after injection and gelation of cellulose solution in the center of double network hydrogel. Cellulose solution was mixed with rhodamine B (pink) to distinguish the inner and outer part of the scaffold. Scale bar = 5 mm. (e) Swelling behavior of cellulose/alginate double hydrogel and cellulose hydrogel at different time points. Error bars show mean ± SD for total *n* = 4. (f) Degradation test of cellulose/alginate double hydrogel and cellulose hydrogel. Error bars show mean ± SD for total *n* = 4. (g) Illustration of in vitro passive release of GDF‐5 protein from cellulose hydrogel and total IVD scaffold. (h) Release profile of GDF‐5 protein from cellulose hydrogel and total IVD scaffold at predetermined time points. Error bars show mean ± SD for total *n* = 6.

In this study, two different functional peptide sequences were modified on polymer side chains to promote the innate regenerative ability via endogenous MSCs recruitment and regulation. MSC homing peptide (SKPPGTSS) was designed and grafted on the side chain of oxidized cellulose to form cellulose‐SKP, whereas cell adhesion peptide (GRGDSP) was immobilized on alginate network to form alginate‐RGD. Together, the synthesized cellulose‐SKP and alginate‐RGD were then used to fabricate cellulose/alginate double network hydrogel. The peptide functionalization and coupling were verified by FT‐IR spectra and ^1^H NMR, respectively (Figures S1 and S2). After epichlorohydrin (ECH) crosslinking, a transparent cellulose‐based hydrogel was formed (Figure 2a). The residual ECH was removed by PBS washing. For cellulose/alginate double network hydrogel, a light‐yellow hydrogel was synthesized by the addition of alginate powder and then the gel was further immersed in CaCl_2_ solution (Figure 2b). Next, the central part of cellulose/alginate double network hydrogel was removed by a biopsy punch, forming a hollow cylinder (Figure 2c). The solution of ECH‐crosslinked cellulose was then injected into the hollow center of the hydrogel, yielding the total IVD scaffold (Figure 2d). SEM was utilized to visualize the microporous structure of the hydrogel (Figure S3). The interconnected porous structure provides a spatial orientation and allows cells to migrate and proliferate during the regeneration.

Due to the presence of hydrophilic polymer chains, hydrogels exhibit high water content, and are responsible for the interactions with water and subsequent volume change upon implantation. Here, the swelling ratio of cellulose and cellulose/alginate DN hydrogel was investigated in different time points (Figure 2e). After immersing in PBS for 24 h, cellulose hydrogel achieved a significant increase in weight change compared with that of DN hydrogel. The degradation profile in PBS was monitored by weight loss as a function of incubation time (Figure 2f). The profile showed an obvious initial degradation kinetics during the first few days of incubation in cellulose hydrogel group. The reason might be attributed to the different crosslinking methods in both hydrogels. In the cellulose/alginate DN hydrogel, the alginate‐calcium ions network served as a “cage” to prevent water from invading the internal hydrogel; thus, decelerating the overall degradation process. On the other hand, the cellulose hydrogel was crosslinked mainly by ester bonds and could be hydrolyzed readily. After 60 days, cellulose hydrogel retained only 20% of the initial mass, while DN hydrogel possessed 55% of residual mass. This in vitro degradation profile may provide an estimation for the long‐term degradation behavior of IVD scaffold after implantation.

Next, we investigated the passive release kinetics of GDF‐5 from the hydrogels by using ELISA to detect the amount of released proteins in the supernatant (Figure 2g). A burst release of approximately 60% of initial GDF‐5 was observed in cellulose hydrogel after 48 h. Thereafter, the release was linear until 168 h and was sustained through the end of the experiment. After 336 h, 85% of GDF‐5 was released into the supernatant. As for total IVD scaffold, it showed a controlled release behavior for a total release of approximately 50% by 336 h (Figure 2h). The slow release of GDF‐5 in total IVD scaffold may be explained by the presence of periphery structure, which hinder the diffusion of bioactive factors. Such prolonged release profile would achieve sustained effect in the following in vivo study.

### 3.2 Mechanical properties of the hydrogels and total IVD scaffold

**FIGURE 3:**
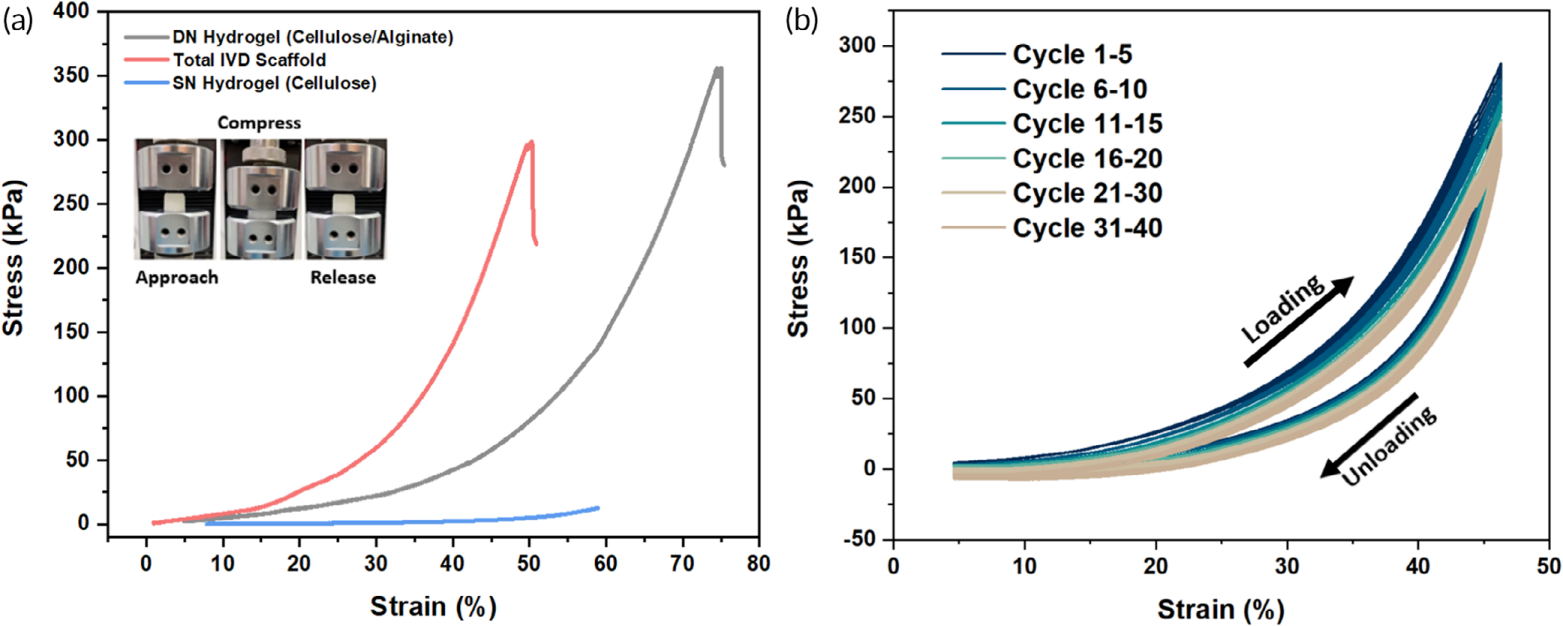
Mechanical properties of the hydrogels and total IVD scaffold. (a) Stress–strain curve of the three types of gel (single network hydrogel, double network hydrogel, and total IVD scaffold) until rupture. Images show the compression test process of the cylinder‐shaped hydrogel. (b) Cyclic test of total IVD scaffold with 40 cycles of loading and unloading

The mechanical property of hydrogels was measured through universal testing machine (UTM). With the addition of alginate, DN hydrogel exhibited a higher mechanical strength and strain under compressive loading, compared with cellulose hydrogel (Figure 3a). An ultimate tensile strength of 351 and 12.7 kPa was measured in DN and single network (SN) hydrogel, respectively. Moreover, at the point of rupture, the elongation of DN hydrogels increased to nearly 75% upon combining with alginate network, presenting considerable enhancement in mechanical properties. The modulus and strain of total IVD scaffold (297 kPa and 55%, respectively) were slightly lower than those of DN hydrogel.  Nonetheless, the IVD scaffold mainly composed of DN hydrogel still showed promising mechanical properties for total disc replacement.

Cyclic test was conducted to further examine the mechanical behavior of total IVD scaffold under repeating loading and unloading (Figure 3b). The area between the loading and unloading curves represents the dissipating energy per unit volume. The larger area represents a higher dissipated energy, suggesting a significant hysteresis during compressive process; while smaller area shows a lower dissipated energy, resulting from full recovery process after unloading. Though the total IVD scaffold showed hysteresis loops after cycles of loading and unloading, there were no considerable changes within 40 cycles. These results indicated that the total IVD scaffold could maintain its integrity and recover to its original shape without permanent deformation after subjecting to cycles of continuous compressive loading and unloading (Movie S1).

### 3.3 Shape memory properties of DN hydrogel

The design strategy for shape memory hydrogel primarily involves two distinct components: permanent crosslink which determines the permanent shape, and reversible crosslink which fixes its temporary shapes. In our DN hydrogel, the covalent bonds in cellulose network may act as permanent crosslink, while the noncovalent bonds in alginate network serve as reversible crosslink.

**FIGURE 4:**
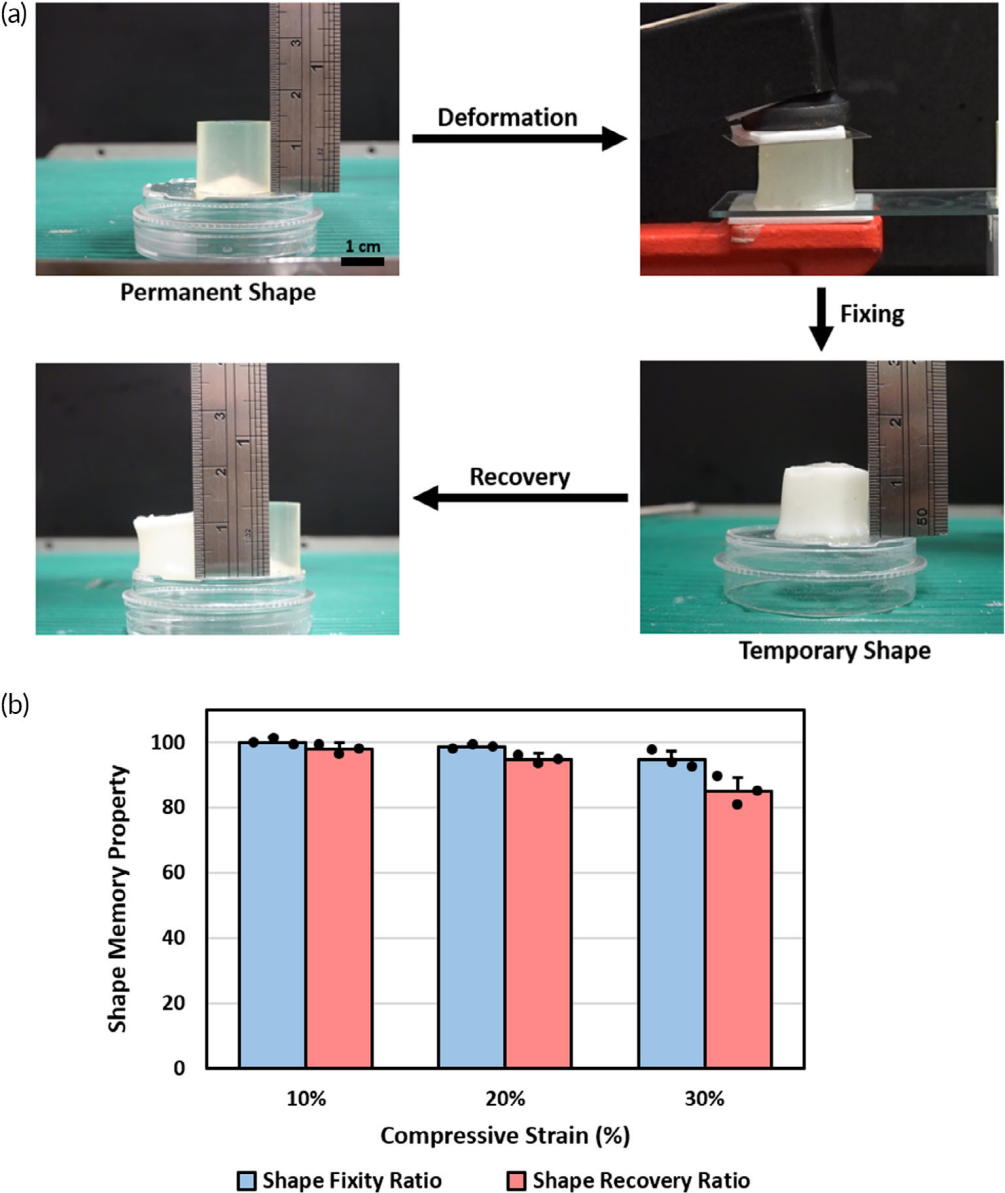
Shape memory property of cellulose/alginate double network hydrogel. (a) Photographs of the shape memory process, including deformation, fixing, and recovery. (b) Results of shape fixity and recovery ratio. Error bars show mean ± SD for total *n* = 3

Owing to the combination of cellulose and alginate network, shape memory property could be observed after the addition of ethylenediaminetetraacetic acid (EDTA) chelating agent. EDTA has been widely used in complex with ions, especially divalent ions, through its hexadentate structure. The immersion of cellulose/alginate double network hydrogel in EDTA solution induces the chelating process, resulting in the removal of calcium ions in the hydrogel. Figure 4a demonstrated the shape memory process of cellulose/alginate double network hydrogel. In the permanent shape state, cellulose/alginate hydrogel (without calcium ions crosslinked) was prepared in advance. Thereafter, the hydrogel was compressively deformed 30% strain and fixed in calcium ion solution. The crosslinked alginate network in hydrogel would fix the deformed shape and impede the elastic deformation by the crosslinking between alginate and Ca^2+^, forming the temporary shape. During recovery state, the addition of EDTA triggered the escape of Ca^2+^ from alginate network, leading to the breakdown of reversible crosslink. After 30 min of immersion, the compressed hydrogel soon recovered to the permanent shape (Movie S2).

To quantify the shape memory behavior of the DN hydrogel more precisely, the parameters of shape fixity and shape recovery ratio were measured (Figure 4b). As the compressive strain of double network hydrogel was set to 10% and 20%, the shape fixity ratio and recovery ratio were over 95%. The two parameters were still higher than 85% even when the compressive strain was increased to 30%. The explanation for slight decrease in the recovery ratio was the buckling of hydrogel network during deformation. Since there was low physical crosslinking density between alginate network with calcium ions, the compressed hydrogels were unable to fully recover to their original shape. Such phenomenon could be adjusted by modulating physical crosslinking density for future clinical requirements.

### 3.4 In vitro controlled signaling regulation of MSCs

To confirm the possibility of using the total IVD scaffold as structural support for injured disc replacement, the cytocompatibility of MSCs when co‐cultured with cellulose and cellulose/alginate DN hydrogel was examined, respectively. The cell viability and cytotoxicity of both hydrogels were evaluated by Live/Dead staining and LDH assay (Figure S4). The Live/Dead staining results showed the biocompatibility of both hydrogels co‐cultured with MSCs. Live MSCs (green fluorescence) were dominant in visualized fields, while dead cells (red fluorescence) were rarely observed after 7 days of incubation. The ratio of dead cells in both cellulose and cellulose/alginate DN hydrogel remained low and had no significant difference comparable to cells cultured in tissue‐culture polystyrene (negative control) after 7 days.

**FIGURE 5:**
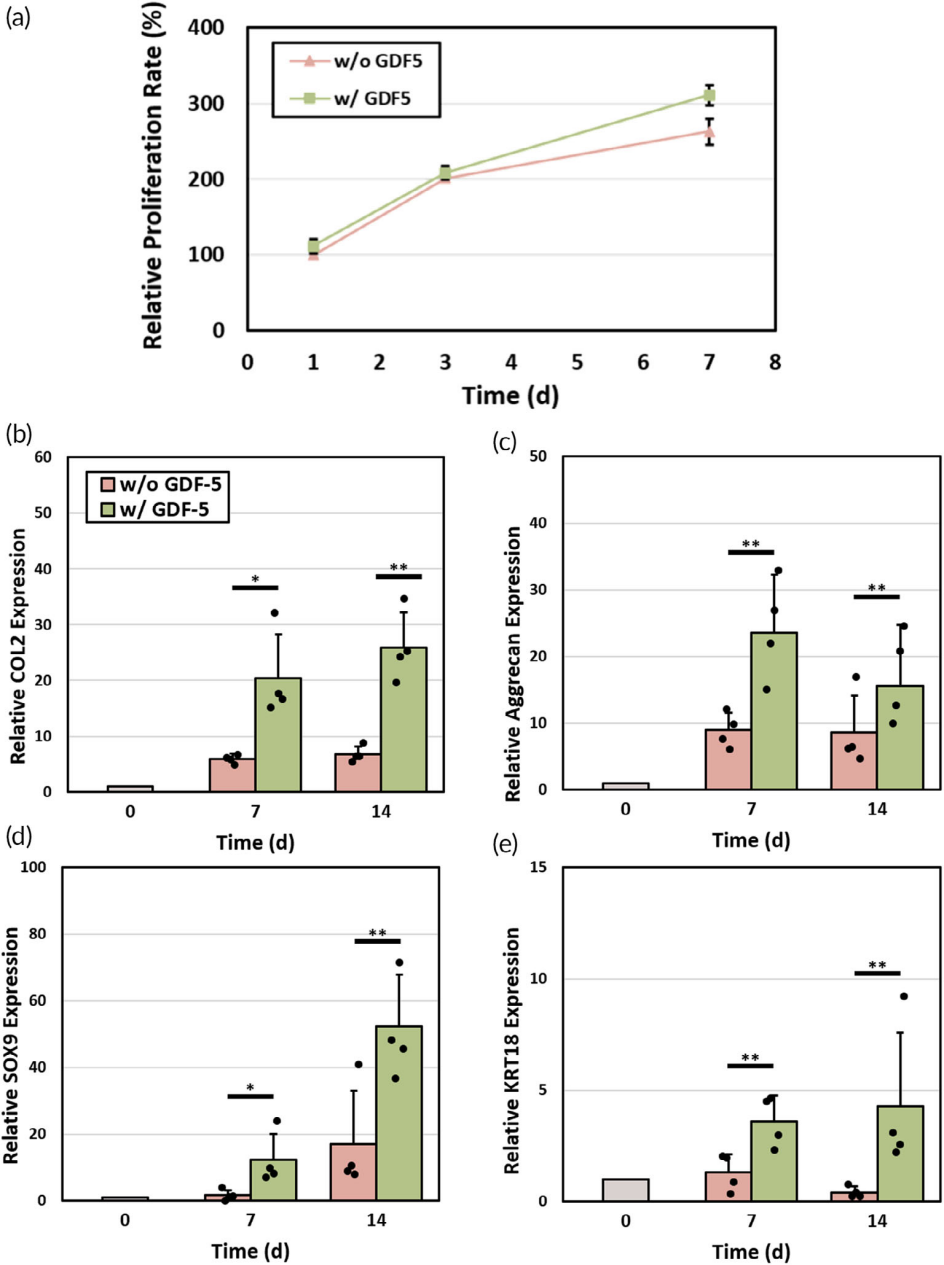
Effect of GDF‐5 on MSC proliferation and differentiation. (a) The relative proliferation rate of MSCs cultured in total IVD scaffolds with and without loaded GDF‐5 protein at different time points. Error bars show mean ± SD for total *n* = 4. (b–e) Gene expression levels of MSCs cultured in total IVD scaffolds with and without GDF‐5 protein at different time points. GAPDH served as housekeeping gene. All genes were normalized to GAPDH levels. Error bars show mean ± SD for total n = 4 (*p < 0.05 and **p < 0.01)

Since MSC proliferation is an important factor in endogenous IVD regeneration, we first evaluated the ability of growth factor GDF‐5 by CCK‐8 assay (Figure 5a). MSCs were co‐cultured with total IVD scaffold either with or without GDF‐5. In GDF‐5 loaded scaffold at day 7, the relative proliferation rate tended to be higher when compared with pristine scaffold group, although this difference was not significant. It is possible that cellular proliferation was not the dominant effect of GDF‐5. To further investigate the differentiation effect of GDF‐5 treatment, quantitative PCR analyses of type II collagen (*COL2*), aggrecan (*ACAN*), *SOX9*, and keratin 19 (*KRT19*) were performed. *COL2* and *ACAN* are important indicators for ECM synthesis and deposition, while *SOX9* and *KRT19* are specific gene markers for chondrogenic and NP‐like phenotype differentiation. The GDF‐5 loaded group exhibited higher expression levels of *COL2* and *ACAN* on day 7 and 14, indicating the promotive effect of GDF‐5 on ECM deposition (Figure 5b, c). Also, *SOX9* gene expression in GDF‐5 loaded group was 7.5‐ and 3.1‐fold higher after 7 and 14 days of culture (Figure 5d). It is worth reporting that the gene expression level of *KRT19* in GDF‐5 loaded group was 2.8‐ and 10.4‐fold higher than that in the group without GDF‐5 treatment on day 7 and 14, suggesting the MSCs were undergoing NP‐like phenotype differentiation (Figure 5e).

Next, collagen and glycosaminoglycans (GAGs) expression were assessed to evaluate ECM decomposition. Picrosirus red and Alcian blue staining were utilized to determine the accumulation of collagen and GAGs, respectively (Figure S5). After 7 days coculture, both Alcian blue and Picrosirus red were strongly stained in GDF‐5 loaded group, which correlated to the increasing gene expression of *COL2* and *ACAN*. Notably, there was minimal staining for type I collagen (yellow) in the group without GDF‐5 loading, indicating the treatment of GDF‐5 could promote the expression of type II collagen instead of type I collagen.

### 3.5 In vitro MSC recruitment and adhesion

**FIGURE 6:**
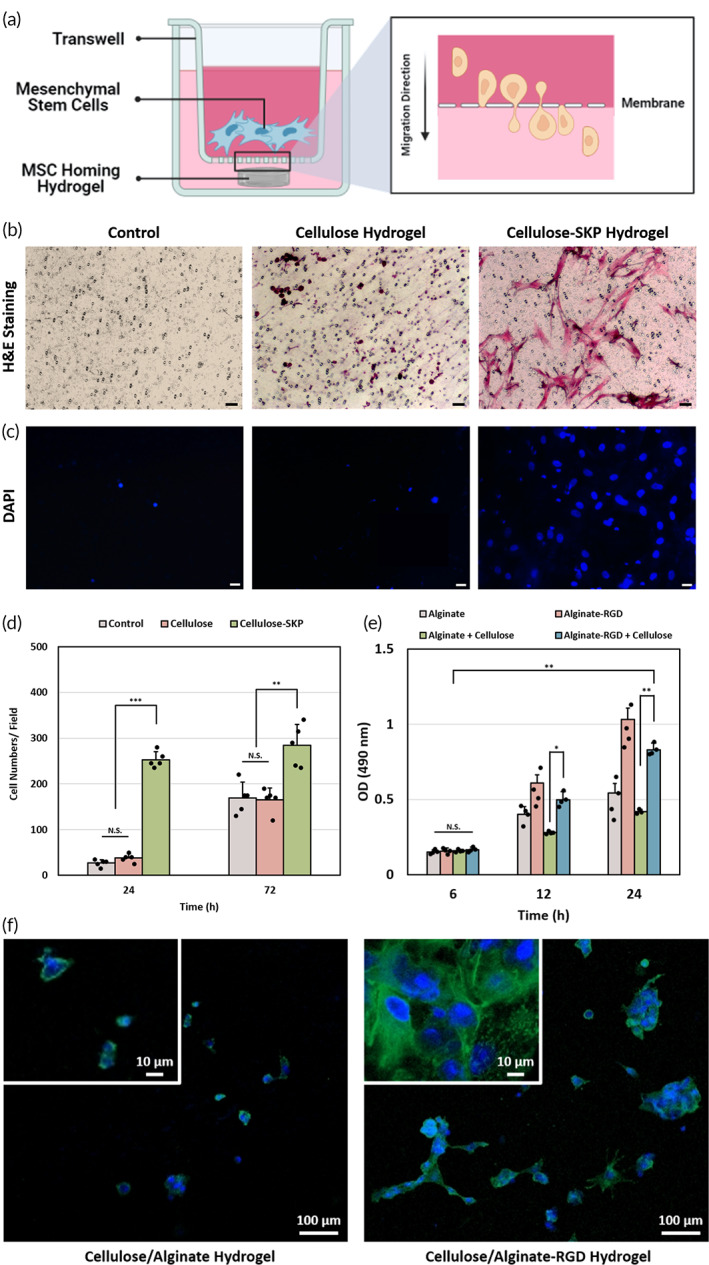
Cell adhesion and migration evaluations via functional peptide motifs. (a) Scheme of the MSC migration model in Transwell system. (b) Migrated MSCs fixed and stained by hematoxylin and eosin (H&E) staining. (c) Migrated MSCs fixed and stained by DAPI. Scale bars = 50 μm. (d) MSC migration after 24 and 72 h reflected by total number of cells in each of the random fields within an insert. Error bars show means ± SD for total *n* = 5 (**p < 0.01, ***p < 0.001, and N.S. represents no significant difference). (e) Cell adhesion property of different groups measured by MTS assay (*p < 0.05, **p < 0.01, and N.S. represents no significant difference) Error bars show means ± SD for total *n* = 4. (f) DAPI (blue) and ACTI‐Stain 488 Phalloidin (green) stained cells were imaged by confocal laser scanning microscopy of two groups (with and without RGD) at 24 h after seeding.

In order to examine the locally directed homing capability of SKP‐modified hydrogel in vitro, we counted the number of migrated MSCs in a Transwell system. This culture system separated the SKP‐modified hydrogel and pristine hydrogel with MSCs to mimic the spatial relations after scaffold implantation similar to the endogenous MSC niches (Figure 6a). The MSC migration from the Transwell insert was determined by H&E staining (Figure 6b). After 24 h of incubation, large amount of MSCs traveled through the membrane and migrated to another side of insert (Figure 6c). This observation indicated that MSCs migration was more prominent in the presence of SKP‐modified hydrogel. The migration assay results were consistent with the fluorescent staining results, suggesting that MSCs readily migrated to the receiving well in SKP group (Figure 6d). Additionally, the MSC homing property was also monitored by directly observing the bottom side of insert via SEM (Figure S6). For SKP group, cellular ECM was observed on the surface of membrane, revealing the cell migration trajectory. All of the above results suggested that MSCs have a high tendency to migrate toward the hydrogel with SKP functional peptide.

Cell adhesive property of RGD grafting hydrogel was examined via MTS assay and observed through confocal laser scanning microscopy after fluorescent staining. For MTS assay, the absorbance value (OD) of each group was proportional to the cell number adhering to the surface of hydrogel (Figure 6e). After 12 and 24 h incubation, hydrogels with RGD modification showed increased cell numbers, indicating RGD‐modified hydrogel could promote early cell attachment. The adhesive MSCs were stained with DAPI and ACTI‐Stain 488 Phalloidin (AF488) in order to directly observe the morphology of cells on hydrogel surface (Figure 6f). After 24 h of cell seeding, number of MSCs attached to the cellulose/alginate‐RGD hydrogel was significantly higher than that of the cellulose/alginate hydrogel group. Moreover, cells on cellulose/alginate‐RGD hydrogel also showed stronger green fluorescence expression in comparison with cellulose/alginate hydrogel, suggesting presence of protruding filopodia and enhanced cell adhesion.

### 3.6 In vivo degenerative disc disease treatment in rat model

**FIGURE 7:**
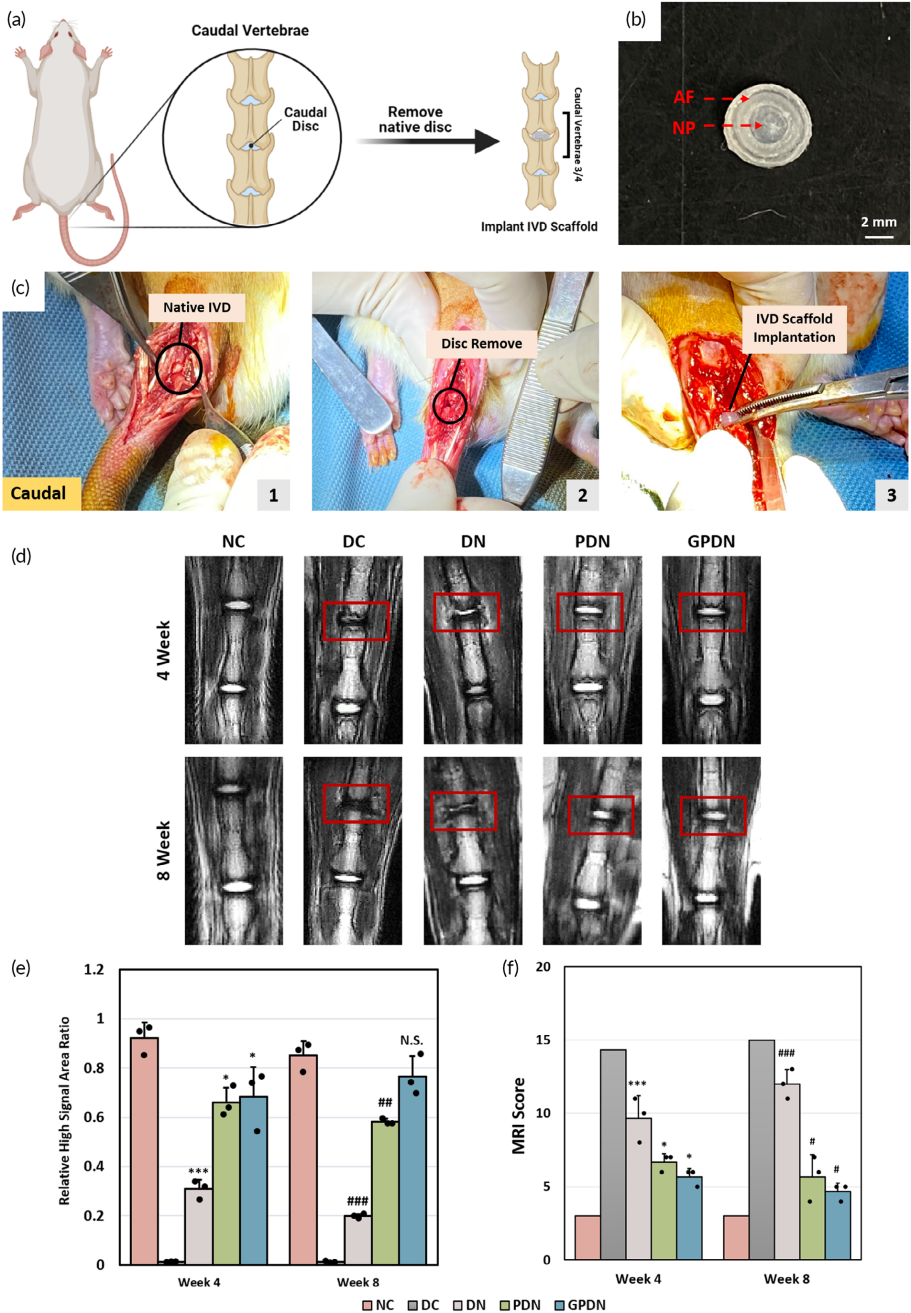
DDD model establishment and monitoring of disc height and scaffold integrity after implantation. (a) Scheme of DDD model. The constructed IVD scaffolds were implanted into the rat caudal spine 3/4 to replace the native caudal disc. (b) Phenotype of implanted IVD scaffolds. (c) Photographs of IVD scaffolds implantation procedure. (1) Surgical process to expose native rat IVD. (2) Caudal disc was removed by rongeur to prepare a disc space. (3) IVD scaffold was implanted into the prepared space. (d) T2‐weighted MRI imaging of the caudal spine (marked with red boxes) of NC, DC, DN, PDN, and GPDN groups at 4 and 8 weeks after implantation. (e) Quantitative measurements of the high signal area in IVD for each experimental condition were obtained at 4 and 8 weeks post implantation. Error bars show mean ± SD for total *n* = 3. (f) Mean MRI scores of targeted discs for each experimental condition. Level data of the MRI images were analyzed using modified Pfirrmann grading system at 4 and 8 weeks. Error bars show means ± SD for total n = 3 (*p < 0.05 and ***p < 0.001 vs. 4 weeks negative control group; ^#^ *p* < 0.05, ^##^ *p* < 0.01 and ^###^ *p* < 0.001 vs. 4 weeks negative control group)

The therapeutic efficacy of total IVD scaffold was evaluated using a rat caudal disc model. The scaffolds were implanted into the caudal spine discs of the rats, between the third and fourth caudal vertebrae (caudal 3/4), to evaluate their long‐term performance (Figure 7a). The shape of the scaffolds was fashioned to fit the space of rat caudal disc (Figure 7b). After removing the attached peripheral connective tissue, native caudal disc was found between two adjacent vertebrae (Figure 7c). Native caudal discs were subsequently removed and the endplate of the vertebrae was carefully preserved. The space was then replaced with injection of prepared scaffolds or saline. Four and eight weeks after scaffold implantation, MRI images were obtained to determine disc height and water content in the IVD scaffolds (Figure 7d). The abbreviation of each experimental group was described in Table S2. The GPDN group showed a fusiform shape with bright signal between the adjacent vertebrae. Similar result was also found in PDN group, indicating that both groups maintained their integrity and water content. Although DN group gave bright signal, the height of scaffold was relatively lower than that of negative control group (NC). On the other hand, no signal was found in DC group, resulting from the absence of any scaffold. To evaluate the disc height and integrity in detail, the area of high signal ratio between implanted scaffold and adjacent native disc was calculated and shown in Figure 7e. The results corresponded to the MRI images. GPDN and PDN groups had similar area ratio with control sham group, indicating stable hydration level in both scaffolds. Moreover, a modified Pfirmann grading score system was utilized to determine the regeneration of IVD. Three categories including the structure of IVD, signal intensity, and the height of disc, were involved in this MRI grading system. The results suggested that both GPDN and PDN groups had better regenerative ability as demonstrated in the MRI image quality and signal intensity when compared with DC and DN groups (Figure 7f).

**FIGURE 8:**
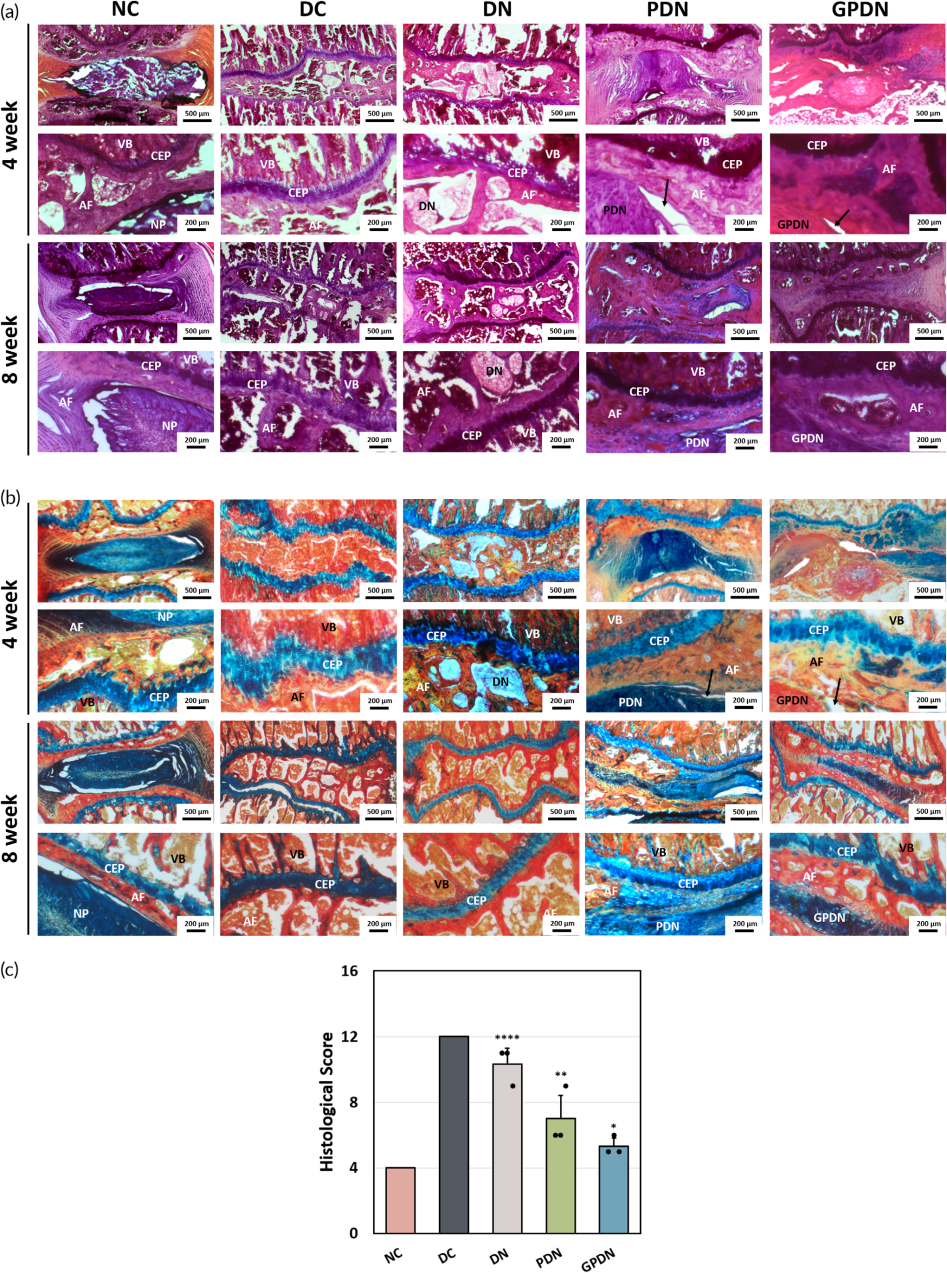
Chondrogenic matrix production, organization, and histological score. (a) Representative images of H&E staining of different groups obtained at 4 and 8 weeks post implantation. (b) Representative images of Alcian blue & Picrosirus red (AbPr) staining of different groups obtained at 4 and 8 weeks post implantation. Black arrows point out small discontinuities in boundary between annulus fibrosus and implanted scaffolds, while red arrows show the newly formed NP tissue (AF, annulus fibrosus; CEP, cartilage endplate; NP, nucleus pulposus; VB, vertebral bodies). (c) Histological scores of five groups evaluated at 8 weeks post implantation. Error bars show mean ± SD for total *n* = 3 (*p < 0.05, **p < 0.01, and ****p < 0.0001 vs. negative control group)

Since the immobilization of RGD cell adhesion peptide on the side chains of alginate network, we hypothesized that the reported total IVD scaffold would integrate with native vertebral bodies and annulus fibrosus. To verify this hypothesis, H&E staining was performed in the disc space (Figure 8a). In the DN group without any peptide modification, considerable discontinuities were observed in the boundaries between DN hydrogel and AF, indicating the poor integration. In contrast, both PDN and GPDN groups showed minimal discontinuitied boundaries between AF and implanted scaffolds. In addition to the integration with adjacent tissue, a critical requirement for a tissue‐engineered IVD scaffold is the production of GAGs and collagen. The amount of collagen and GAGs deposition was observed by conducting AbPr staining (Alcian blue stained for GAGs and Picrosirus red stained for collagen) (Figure 8b). The disc space in DC group was collapsed and no GAGs in NP region was observed due to the removal of native disc. Similar results were found in DN group despite the implantation of DN hydrogel in disc space, suggesting pristine hydrogel had little effect in producing GAGs. On the other hand, blue staining was readily observed in both PDN and GPDN groups within NP region, revealing that incorporation of stem cell homing peptide and GDF‐5 successfully induced GAGs secretion. As shown in Figure 8c, DC and DN groups showed considerable differences compared with NC group, while PDN and GPDN groups showed better histological scores than DC group (the lower, the better). These results indicated that both PDN and GPDN groups possessed disc reparative ability.

**FIGURE 9:**
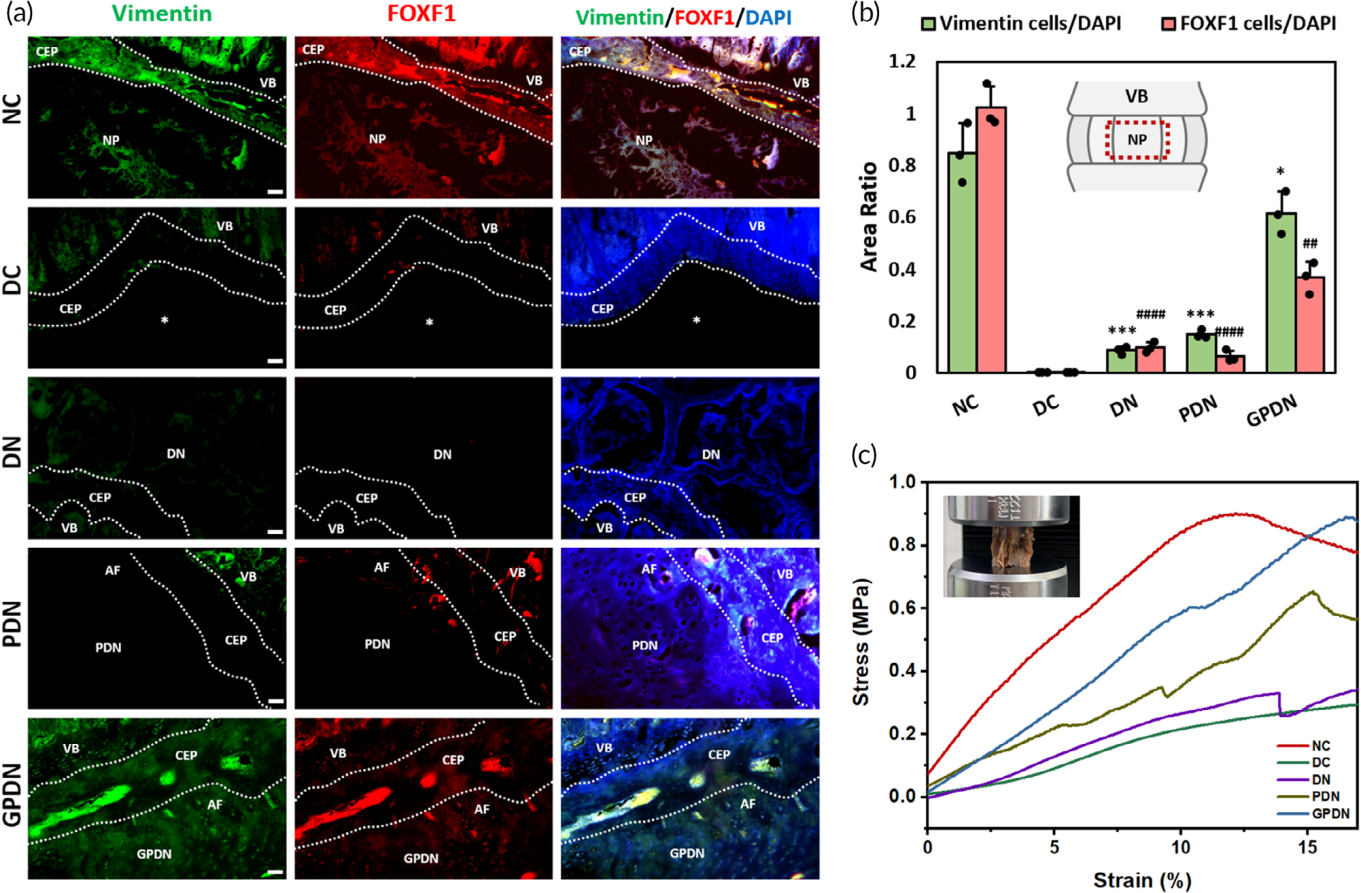
NP‐like phenotype induction and mechanically functional tissue production by different IVD scaffolds. (a) NP‐like phenotype induced by different IVD scaffolds after 8 weeks of implantation. Intervertebral disc and vertebral body sections were stained with Vimentin immunoreactivity (green), FOXF1 NP‐like cell staining (red), and merged with DAPI nucleus staining (blue) for different groups. Dotted lines represent boundaries between each part of IVD; star signs represent the empty space caused by native disc discectomy. Scale bars = 50 μm (AF, annulus fibrosus; CEP, cartilage endplate; NP, nucleus pulposus; VB, vertebral bodies). (b) Vimentin and FOXF1 protein expression were quantified by the area of Vimentin‐positive and FOXF1‐positive cell signals divided by the area of DAPI signals. Red square outlines the measuring area. Error bars show mean ± SD for total *n* = 3 (*p < 0.05, ***p < 0.001, and ****p < 0.0001 vs. negative control in Vimentin group; ^##^ *p* < 0.01, and ^####^ *p* < 0.0001 vs. negative control in FOXF1 group). (c) Plots of uniaxial compressive stress versus strain for different groups after 8 weeks of implantation

NP‐specific proteins, vimentin, and forkhead‐box F1 (FOXF1), were utilized to examine the differentiation of stem cells near regenerated tissue in the 8 weeks (Figure 9a). As one of the major elements of cytoskeleton, vimentin played a critical role in cell division as well as the dynamic functions of NP cells. On the other hand, FOXF1 was identified as a healthy‐NP‐specific marker which involved in regulating cell growth and proliferation. The expression of these two proteins was down regulated during disc degeneration; thus, they could be used as markers to determine MSC differentiation and function (Figure S7). After 4 weeks of implantation, no vimentin‐positive cells could be found in the disc space in DC group. However, when incorporating GDF‐5 in the scaffold (GPDN group), vimentin expression was significantly enhanced compared with that of the DN and PDN groups. Similar results were observed when testing the FOXF1 expression. The number of FOXF1‐positive cells found in scaffold region after the addition of GDF‐5 was considerably higher than those in scaffolds without GDF‐5. Immunohistochemical staining showed that NP‐related protein expression was co‐localized with DAPI labeled cells within the disc space region in GDPN group. Quantification of area ratios of vimentin‐ and FOXF1‐positive cells to DAPI signals showed that scaffold implantation with GDF‐5 incorporation had a higher area ratio when compared with other groups, suggesting its ability to induce MSC differentiation toward NP‐like phenotype (Figure 9b). The synergistic effect between functional peptide modifications and growth factor encapsulation could be further proved by prominent differentiated MSCs into NP cells, which expressed related markers.

The capability to maintain integrity and sustain uniaxial compressive forces is the key function of artificial disc. To verify how the reported total IVD scaffolds reinforce this function after implantation, the biomechanical properties of motion segments were analyzed 8 weeks after replacement (Figure 9c). When the strain ratio fell <10%, the strength for NC group was the highest among all experimental groups. Owing to the discectomy disc and degradation of scaffold, relatively lower strength was noticed in DC and DN groups when compared with that in PDN and GPDN groups. Interestingly, a decline of compressive stress emerged in NC group as the strain ratio increased to approximately 15%, suggesting the collapse of native disc. Identical results could be found in GPDN group, showing the implants were mechanically comparable and functional within the motion segment and provided enough support under excess loadings.

## 4 DISCUSSION

In this study, the fabricated cellulose‐alginate DN hydrogel (~350 kPa) showed enhanced elastic modulus compared with that of cellulose hydrogel (~20 kPa) (Figure 3a). This result suggests that mechanical properties of DN hydrogel are profoundly modified by the existence of second polymer network (alginate). The presence of alginate network might act as sacrificial bonds, in that the physical crosslinks between alginate and Ca^2+^ are impacted prior to cellulose network when encountering excess loading. As a result, to ensure the integrity of scaffold under constant loading, the DN hydrogel acts as the AF part of the scaffold. The synthesized DN hydrogels displayed stable swelling behavior compared with that of cellulose hydrogel. By incubating hydrogels in PBS for 2 months, the degradation profile suggests that cellulose‐alginate DN hydrogel exhibits a prolonged degradation process, attributing to the strong entanglement between cellulose and alginate networks. The entangled networks as well as the combination of chemical and physical crosslinking slows down the hydrolysis rate of ester bonds in DN hydrogel. Despite the relatively rapid degradation, growth factors loaded in cellulose hydrogel is a promising strategy to deliver bioactive factors. In order to prevent burst release of GDF‐5 in cellulose hydrogel, surrounding cellulose‐alginate DN hydrogel was designed and included in the scaffold (Figure 2g), which minimized the cumulative GDF‐5 release from 60% to 15% in 48 h. In short, the different degradation rates of hydrogels demonstrate a sustained release of GDF‐5, achieving long‐term growth factor treatment effect.

Shape memory DN hydrogel made up of stable cellulose network and switchable alginate network was also reported (Figure 4a). The crosslink between alginate network and Ca^2+^ as well as the entanglement between two polymer networks in the DN hydrogel work together to enhance the recovery from temporary deformations. Furthermore, the timeframe of recovery process within 30 min induced by EDTA chelation would be suitable for the implantation surgical procedure. The concentration of EDTA that we used in the shape memory experiment was around 10–20 mM. According to the previous literature, EDTA levels above 20 mM concentration may have cytotoxic concerns when added to biological samples.^28^, ^29^ Of note is that the exposure time of EDTA can be controlled in our study to minimize the cytotoxic effect. From the Movie S2, it should be noted that the hydrogel was recovered to the permanent shape within 1 min, indicating that the EDTA injecting time could be decreased to <5 min. Furthermore, a lower concentration of EDTA solution could be considered when inducing the shape recovery process, thus the concern for the EDTA cytotoxic issue was minimized.

One of the important strategies for regulating MSCs is the delivery of growth factor GDF‐5 in cellulose hydrogel, which is achieved by the NP part of the scaffold. Although the cell number of MSCs co‐cultured with GDF‐5‐containing hydrogel did not show significantly increase when compared with negative control (Figure 5a), it has been proved that GDF‐5 could induce MSC differentiation toward chondrocyte and NP‐like phenotype. For chondrogenic gene *SOX9* and NP‐specific gene *KRT19*, both relative gene expression levels in GDF‐5 loaded group were considerably higher than those of the group without GDF‐5 treatment (Figure 5b–e). This part of data was also supported by Alcian blue and Picrosirus red staining with the deposition of proteoglycans and collagen, respectively. The above results suggest that the hydrogel microenvironment and bioactive molecule GDF‐5 played a synergistic effect on enhancing MSCs differentiation toward NP cells at both transcriptional and translational levels.

The ultimate goal in developing peptide functionalized DN hydrogel is to enhance endogenous MSCs homing and induce differentiation to regenerate injured IVD. The hydrogel with functional SKP peptide modification could stimulate the chemotaxis of MSCs in 3 days (Figure 6d). Compared with pristine hydrogel, hydrogel grafted with SKP peptide was able to significantly augment MSC homing. The reason underlying this phenomenon is the release of SKP peptide, resulting from the breakdown of dynamic imide bonds between peptide and hydrogel. The free SKP peptide then acts as a chemoattractive agent, forming a gradient for MSC chemotaxis, followed by MSC migration toward hydrogel.

To evaluate the regenerative potentials in vivo, healthy IVD in rat caudal spine was replaced by different scaffolds. The reason for choosing the model is mainly due to repeatability of the surgery, ease of surgical process, and the relatively low level of stress exposure on caudal discs.^30^ The constructed IVD scaffolds included functional peptides and growth factor were inserted into the caudal spine of athymic rats to study their function (Figure 7c). The reported challenges for total disc replacement are: (1) the formation of IVD‐related tissue in the disc space,^31^ (2) the integration with adjacent vertebrae,^32^ and (3) maintaining the scaffold integrity to withstand the constant mechanical loading in disc space. The implanted total IVD scaffold was demonstrated to meet all three requirements in rat caudal disc with the help of functional peptide modification and the encapsulation of growth factor GDF‐5. This study provided unique evidence that the total IVD scaffold has great potential to replace the injured IVD in the spine. It is interesting to note that a previous research has reported distinct IVD degenerative changes and fibrotic healing in response to IVD injury between sexes.^33^ Correlational network analyses showed that males demonstrated clear relationships between injury, structural IVD degeneration, and mechanical allodynia, while females did not. Thus, subtle sex differences in the spine likely interact with nervous system differences. Another research study also pointed out that pain transduction and the development of chronic pain differ between males and females.^34^ Female exhibited increased sensitivity to nerve root injury, suggesting sex differences may be present in an IVD degeneration‐related pain model. Insights from the above works suggest that it is necessary to treat female and male animals as distinct cohorts. Further, there is also need for future work on low back pain models which examines how these spine and nervous system relationships may shift over time and across pain modalities.

A hydrated scaffold was observed within the disc space, maintaining the overall shape and disc height after 8 weeks of implantation under MRI inspection (Figure 7d). The integration with neighboring tissue could be observed in H&E staining results (Figure 8a). With the help of RGD peptide, continuous boundaries between scaffolds and vertebral bodies were found in both PDN and GPDN groups, while discontinuous boundaries were observed in DN group. Moreover, the production of proteoglycans and collagen in GPDN group was similar to healthy caudal disc, demonstrating that MSCs were successfully recruited to disc space and produced functional tissue de novo (Figure 8b). The histological score was calculated by evaluating the morphology of AF, matrix and cellularity of NP, and the border between AF and NP (Figure 8c). After 8 weeks implantation, we found that the scores in GPDN group were relatively lower than that in DC group, suggesting the effect of total IVD scaffold in attenuating IVD degeneration. Additionally, since the GDF‐5 loaded IVD scaffold supported the differentiation of MSCs into NP cells in vitro, the ability of total IVD scaffold to induce MSCs differentiation in vivo was investigated. The incorporation of GDF‐5 led to the up‐regulation of FOXF1 and vimentin within NP region, two proteins that are considered to be associated with NP phenotype (Figure 9a). These findings revealed that peptide‐functionalized scaffold and GDF‐5‐abundant environment have a synergistic effect on supporting the retention, integration, and differentiation of recruited cells. Moreover, one of the major capability of IVD is to sustain axial loading^35^; thus, we verified the mechanical strength of implanted scaffolds after 8 weeks (Figure 9c). Intact motion segments, including the implanted scaffolds and adjacent vertebrae bodies, were examined. Motion segments in GPDN scaffold had similar modulus as the native IVD within 10% of compressive strain, while the other three groups: DC, DN, and PDN groups, had relatively low elastic modulus. This might be due to the collapse of the hydrogels under excess loading and the lack of functional tissues formed in the disc space.

For future perspective, despite the successful implantation of the reported total IVD scaffold in caudal disc space, it is still different from actual clinical applications due to the differences between rat caudal disc and human lumbar disc. Since human beings are bipedal animals with upright standing, the IVD bears more axial loading and would require higher mechanical properties. Meanwhile, considering the larger size and relatively lower permeability of human IVD, the poor nutritional supply and fibrotic bioenvironment in disc space will potentially be a detrimental issue in human degenerative disc disease. A tissue engineered cell/scaffold composite should possess a comparable mechanical strength with nature disc tissue to increase the longevity of implant. To address limitations, translating the IVD scaffold to rat lumbar disc or larger animal models will be our next follow‐up study. In spite of these constraints, this study still introduces the formation of functional tissues in disc space by recruiting endogenous MSCs and inducing the differentiation of MSCs in vivo. The findings herein provide strong support for the development of functional IVD implants and evidence that the reported total IVD scaffold can serve as a promising candidate for future applications in IVD regeneration as well as for other biomedical applications in tissue engineering.

## 5 CONCLUSIONS

In this study, we have successfully developed an IVD‐mimicked and peptide‐functionalized scaffold using cellulose/alginate DN hydrogel for annulus fibrosus (AF) layer and cellulose hydrogel for nucleus pulposus (NP) layer, respectively. The usage of DN structure promotes the mechanical properties of implanted IVD scaffold, which is essential for preventing further damage by adjacent tissue due to mechanical mismatch. Moreover, through the presence of periphery structure, the IVD scaffold allows the local preservation and sustainable release of GDF‐5 growth factor in response to hydrogel degradation. The trigger of shape recovery process by addition of chelating agent enables the scaffold implantation to be done without intense and complicated surgical procedure. In cytocompatibility assessment, the composition of IVD scaffold did not show any toxicity to MSCs when incubated with hydrogel matrix. GDF‐5 released from the IVD scaffold retained its bioactivity for MSC differentiation, as determined by qPCR. The chemotactic migration of MSCs toward the injured site was significantly augmented by the release of SKP peptide, while the RGD peptide‐grafted hydrogel provided platform for the enhancement of cell survival as well as proliferation. In the study of rat caudal disc degeneration model, implantation of structure support through IVD scaffold together with the local delivery of GDF‐5 and functional peptide sequences synergistically retarded the degeneration rate and subsequently enhanced functional recovery. These results demonstrate a potential to control the delivery of bioactive factors while facilitating the chemotaxis and proliferation of endogenous MSCs for IVD regeneration. The findings herein provide support for the development of functional IVD implants and evidence that the challenges associated with artificial disc may be overcome in the near future.

## AUTHOR CONTRIBUTIONS

**Chia‐Yu Ho:** Conceptualization (equal); data curation (lead); formal analysis (lead); investigation (equal); methodology (lead); software (lead); validation (lead); writing – original draft (lead). **Chen‐Chie Wang:** Conceptualization (equal); data curation (supporting); formal analysis (supporting); investigation (supporting); methodology (supporting); resources (supporting); supervision (equal); validation (supporting); visualization (supporting); writing – review and editing (supporting). **Tsung‐Chiao Wu:** Conceptualization (supporting); data curation (supporting); formal analysis (supporting); investigation (supporting); methodology (supporting); supervision (supporting); validation (supporting); visualization (supporting); writing – review and editing (supporting). **Chen‐Hsiang Kuan:** Conceptualization (supporting); data curation (supporting); investigation (supporting); project administration (equal); resources (supporting); validation (supporting); visualization (equal); writing – review and editing (equal). **Yu‐Chung Liu:** Conceptualization (supporting); data curation (equal); formal analysis (equal); methodology (equal); software (equal); validation (equal); writing – original draft (supporting). **Tzu‐Wei Wang:** Conceptualization (lead); funding acquisition (lead); investigation (equal); methodology (supporting); project administration (lead); resources (lead); supervision (lead); validation (lead); writing – original draft (lead); writing – review and editing (lead).

## CONFLICT OF INTEREST

The authors declare that they have no known competing financial interests or personal relationships that could have appeared to influence the work reported in this paper.

### PEER REVIEW

The peer review history for this article is available at https://publons.com/publon/10.1002/btm2.10447.

## Supporting information


**FIGURE S1.** Characterization of cellulose‐SKP.
**FIGURE S2**. Characterization of alginate‐RGD.
**FIGURE S3**. SEM observation of cellulose and cellulose/alginate double network hydrogel.
**FIGURE S4**. Cell viability and cytotoxicity of mesenchymal stem cells (MSCs) co‐cultured with cellulose hydrogel and cellulose/alginate double network hydrogel (DN hydrogel).
**FIGURE S5**. Protein expression of MSCs cultured in IVD scaffolds with and without GDF‐5 protein.
**FIGURE S6**. SEM observation of MSC migration at 24 h.
**FIGURE S7**. NP‐like phenotype differentiation induced by different IVD scaffolds after 4 weeks of implantation.
**FIGURE S8**. Phenotype of hydrogel samples and IVD scaffolds.
**FIGURE S9**. Magnetic resonance imaging (MRI) of rat caudal disc.
**TABLE S1**. Primers used in quantitative RT‐PCR.
**TABLE S2**. Description of each animal experimental group.
**TABLE S3**. Modified Pfirrmann grading for lumbosacral disc degeneration.
**TABLE S4**. Histological grading scale based on four categories of degenerative changes.

**MOVIE S1** Compression test of total IVD scaffold

**MOVIE S2** Shape recovery process of double network hydrogel

## Data Availability

The raw data required to reproduce these findings are available to download as requested. The processed data required to reproduce these findings are available to download as requested.
