# Supplementary material for: Peptide‐functionalized double network hydrogel with compressible shape memory effect for intervertebral disc regeneration
Source: Bioeng Transl Med. 2022 Nov 17;8(2):e10447. doi: 10.1002/btm2.10447 (PMC10013763; doi:10.1002/btm2.10447)
Supplement: Supplementary file 1 — FIGURE S1. Characterization of cellulose‐SKP. FIGURE S2. Characterization of alginate‐RGD. FIGURE S3. SEM observation of cellulose and cellulose/alginate double network hydrogel. FIGURE S4. Cell viability and cytotoxicity of mesenchymal stem cells (MSCs) co‐cultured with cellulose hydrogel and cellulose/alginate double network hydrogel (DN hydrogel). FIGURE S5. Protein expression of MSCs cultured in IVD scaffolds with and without GDF‐5 protein. FIGURE S6. SEM observation of MSC migration at 24 h. FIGURE S7. NP‐like phenotype differentiation induced by different IVD scaffolds after 4 weeks of implantation. FIGURE S8. Phenotype of hydrogel samples and IVD scaffolds. FIGURE S9. Magnetic resonance imaging (MRI) of rat caudal disc. TABLE S1. Primers used in quantitative RT‐PCR. TABLE S2. Description of each animal experimental group. TABLE S3. Modified Pfirrmann grading for lumbosacral disc degeneration. TABLE S4. Histological grading scale based on four categories of degenerative changes. [file BTM2-8-e10447-s001.docx]

Supporting Information

**Peptide-functionalized Double Network Hydrogel with Compressible Shape Memory Effect for Intervertebral Disc Regeneration**

*Chia-Yu Ho^1^, Chen-Chie Wang^2,3^, Tsung-Chiao Wu^2^, Chen-Hsiang Kuan^4, 5, 6^, Yu-Chung Liu^1^, Tzu-Wei Wang^1*^*

^1^Department of Materials Science and Engineering, National Tsing Hua University; Hsinchu, Taiwan

^2^Department of Orthopedic Surgery, Taipei Tzu Chi Hospital, Buddhist Tzu Chi Medical Foundation; New Taipei City, Taiwan

^3^Department of Orthopedics, School of Medicine, Tzu Chi University; Hualien, Taiwan

^4^Division of Plastic Surgery, Department of Surgery, National Taiwan University Hospital; Taipei, Taiwan

^5^Graduate Institute of Clinical Medicine, College of Medicine, National Taiwan University; Taipei, Taiwan

^6^Research Center for Developmental Biology and Regenerative Medicine, National Taiwan University, Taipei, Taiwan

**Corresponding author**

Tzu-Wei Wang, Ph.D.

Professor

Department of Materials Science and Engineering, National Tsing Hua University

101, Section 2, Kuang-Fu Road, Hsinchu, Taiwan 30013, R.O.C.

Tel: +886-3-5715131 ext. 33856

Email address: twwang@mx.nthu.edu.tw

**Other Supplementary Materials for this manuscript include the following:**

Figure S1 . Characterization of cellulose-SKP.

Figure S2. Characterization of alginate-RGD.

Figure S3. SEM observation of cellulose and cellulose/alginate double network hydrogel.

Figure S4. Cell viability and cytotoxicity of mesenchymal stem cells (MSCs) co-cultured with cellulose hydrogel and cellulose/alginate double network hydrogel (DN hydrogel).

Figure S5. Protein expression of MSCs cultured in IVD scaffolds with and without GDF-5 protein.

Figure S6. SEM observation of MSC migration at 24 hours.

Figure S7. NP-like phenotype differentiation induced by different IVD scaffolds after 4 weeks of implantation.

Figure S8. Phenotype of hydrogel samples and IVD scaffolds.

Figure S9. Magnetic resonance imaging (MRI) of rat caudal disc.

Table S1. Primers used in quantitative RT-PCR.

Table S2. Description of each animal experimental group.

Table S3. Modified Pfirrmann grading for lumbosacral disc degeneration.

Table S4. Histological grading scale based on four categories of degenerative changes.

Movie S1. Compression test of total IVD scaffold

Movie S2. Shape recovery process of double network hydrogel


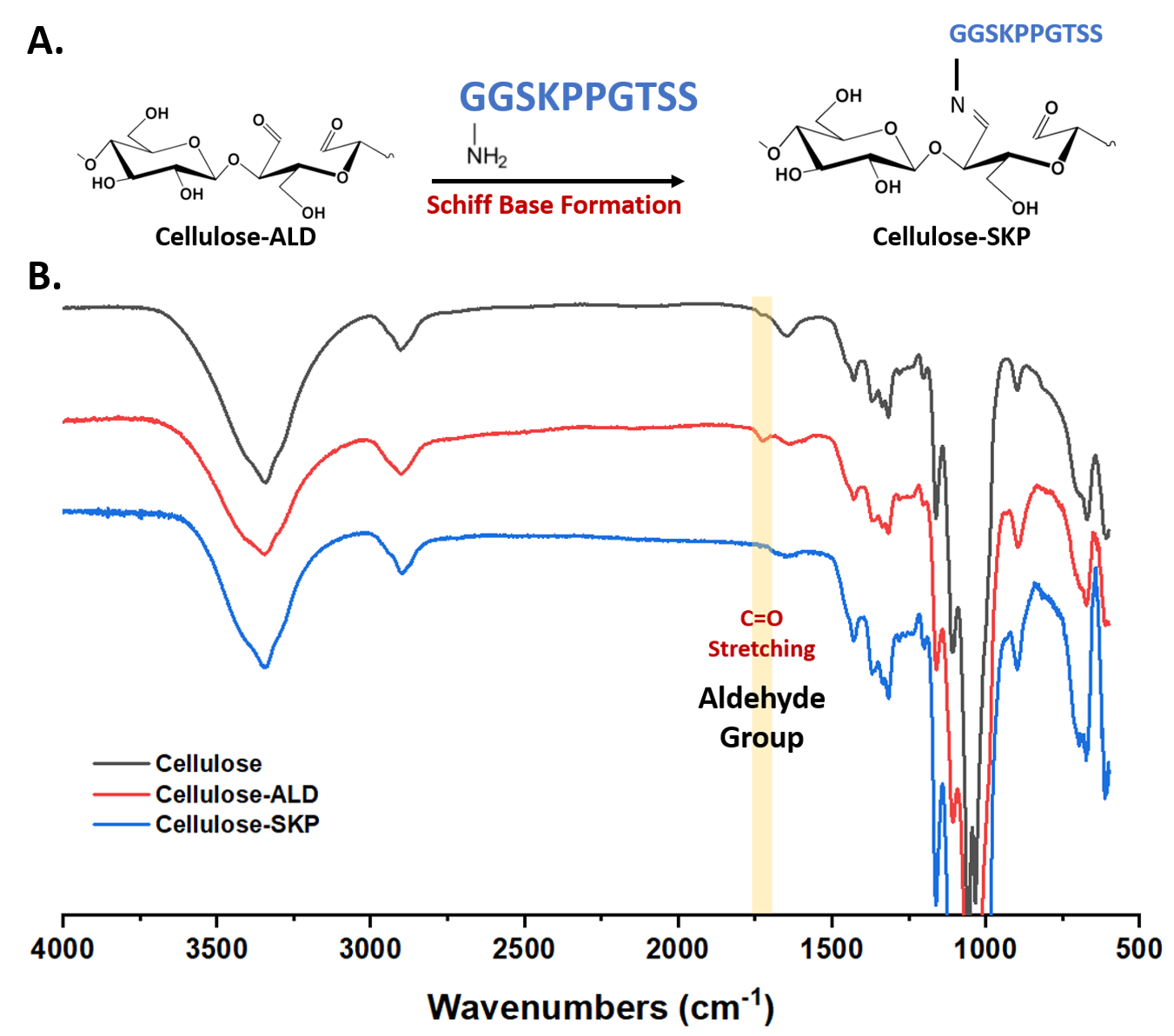


Figure S1. Characterization of cellulose-SKP. (A) Schiff base formation between SKP peptide and cellulose-ALD. (B) FT-IR characterization of cellulose (black), cellulose-ALD (red), and cellulose-SKP (blue).


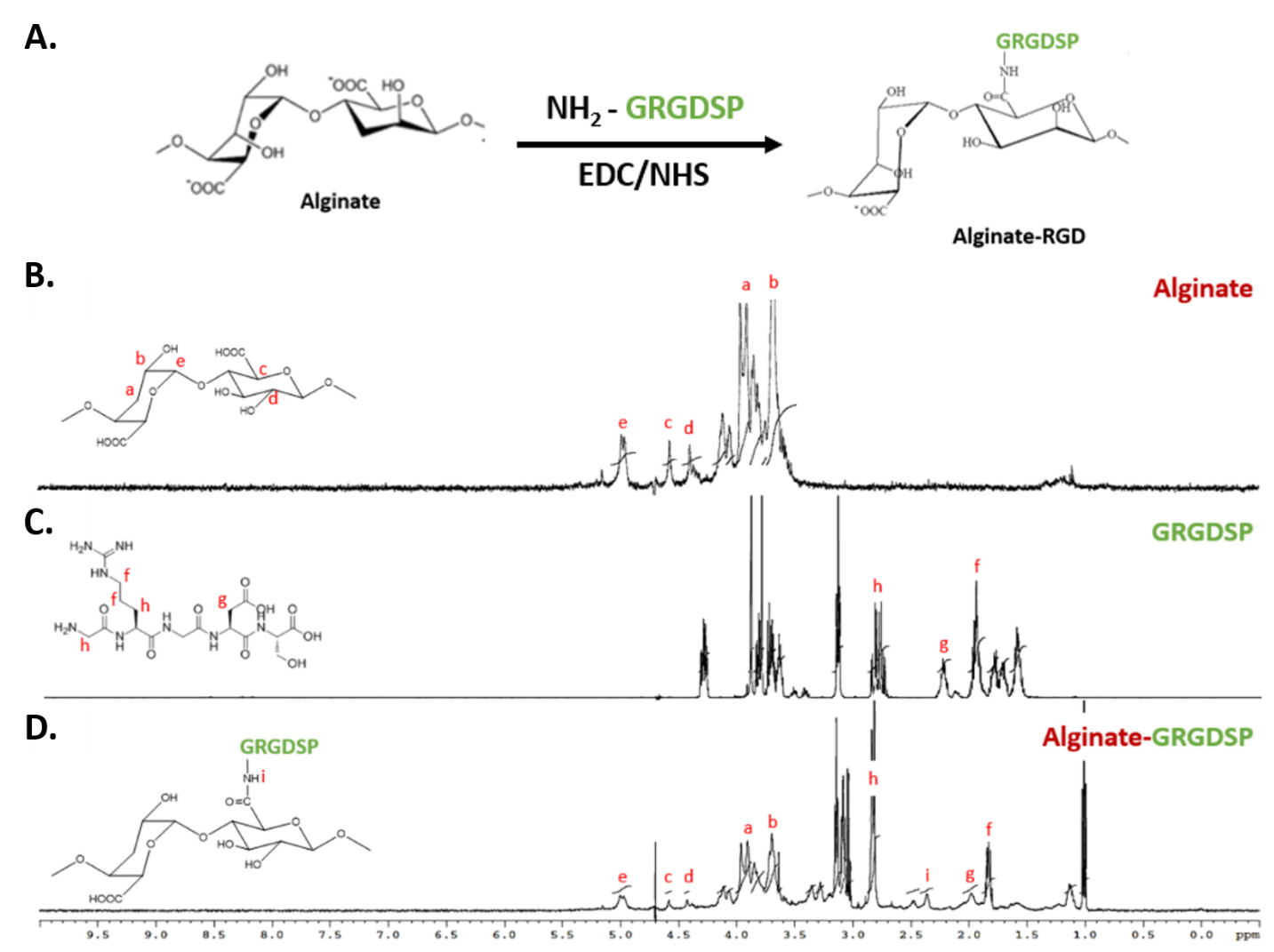


Figure S2. Characterization of alginate-RGD. (A) RGD peptide conjugated on alginate side chain through EDC/NHS chemical reaction. (B)-(D) Peptide conjugation by coupling with alginate was analyzed by 1H NMR spectra. (B) Pure alginate. (C) RGD peptide. (D) Alginate-RGD.


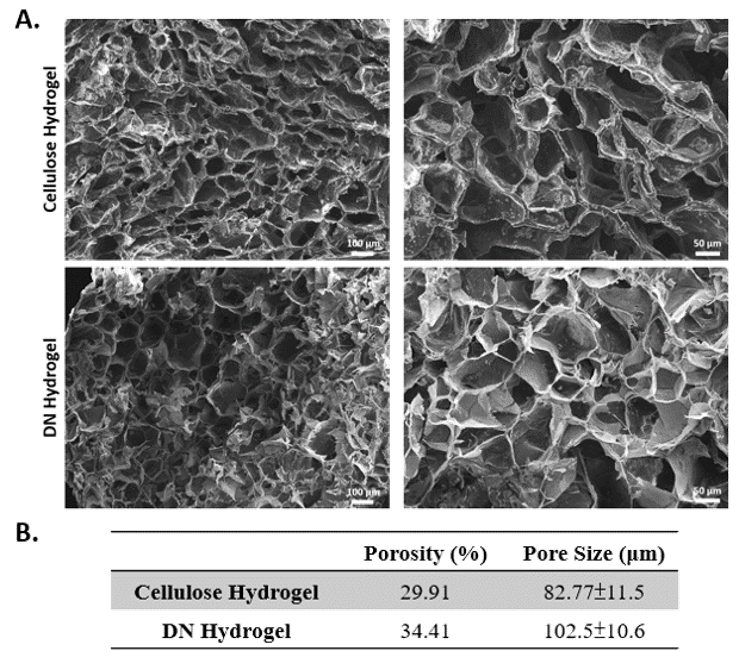


Figure S3. SEM observation of cellulose and cellulose/alginate double network hydrogel. (A) SEM microphotograph of the porous structure of two hydrogels under different magnifications. (B) Degree of porosity and pore size determined by Image J software. Means ± S.D. for total n=4.


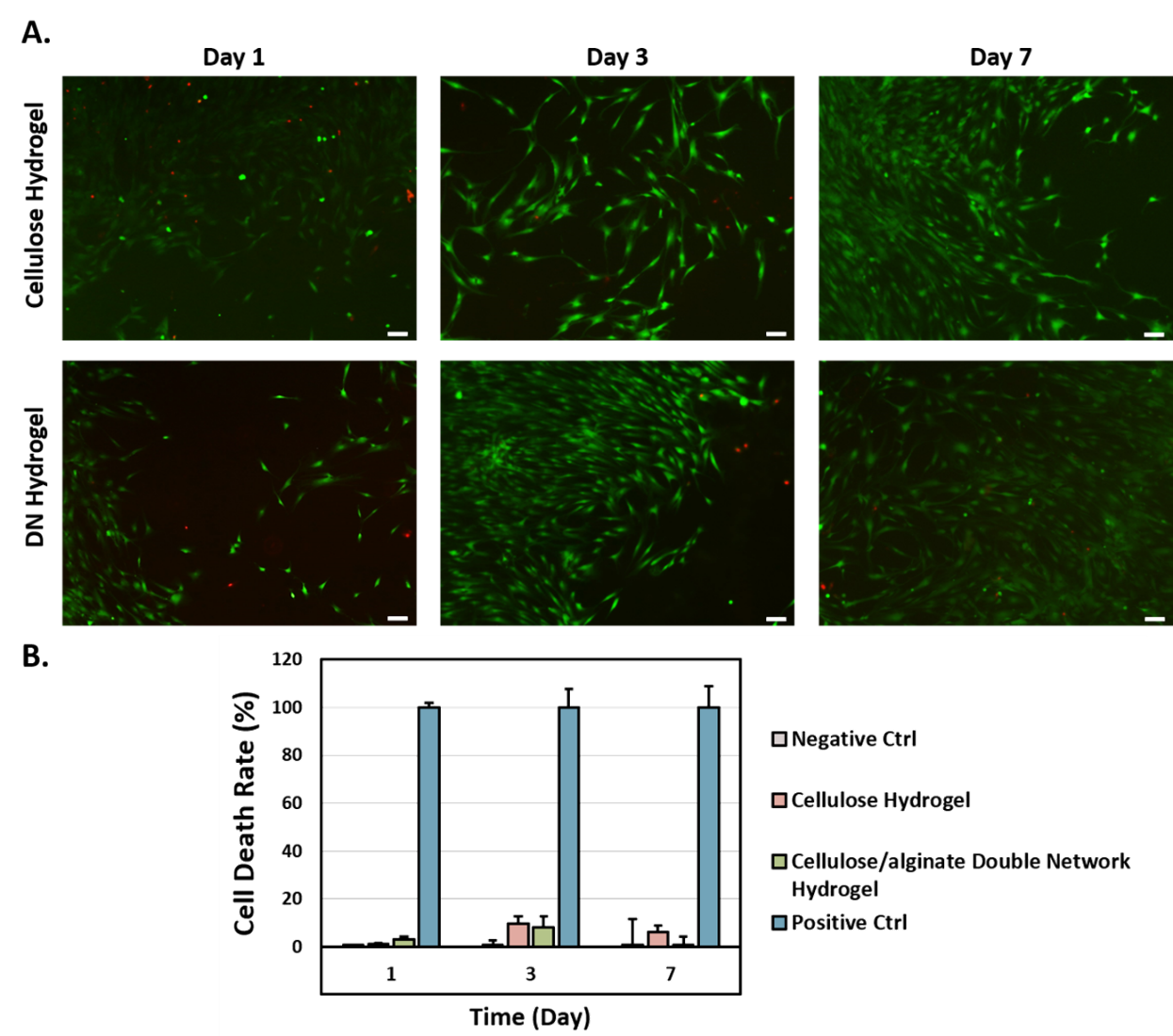


Figure S4. Cell viability and cytotoxicity of mesenchymal stem cells (MSCs) co-cultured with cellulose hydrogel and cellulose/alginate double network hydrogel (DN hydrogel). (A) Live/dead staining and (B) LDH assay after 1,3 and 7 days of incubation. Scale bars = 100 μm. Error bars show means ± S.D. for total n=4.


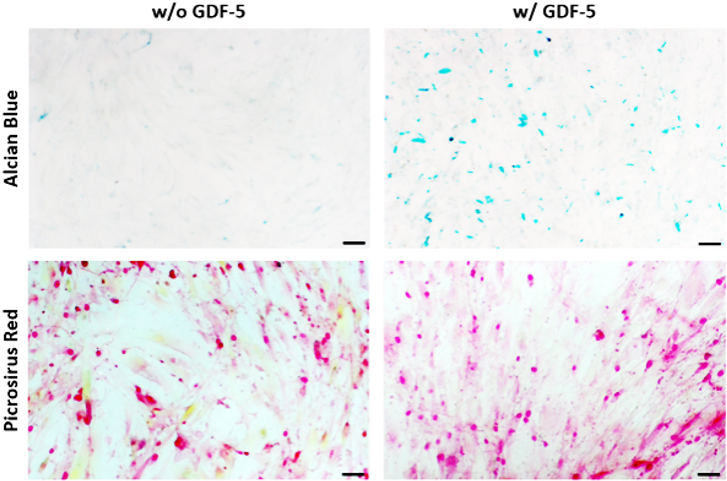


Figure S5. Protein expression of MSCs cultured in IVD scaffolds with and without GDF-5 protein. Representative images of Alcian blue and Picrosirus red staining of different groups obtained at 7 days after incubation. Scale bars = 50 μ­­­m.


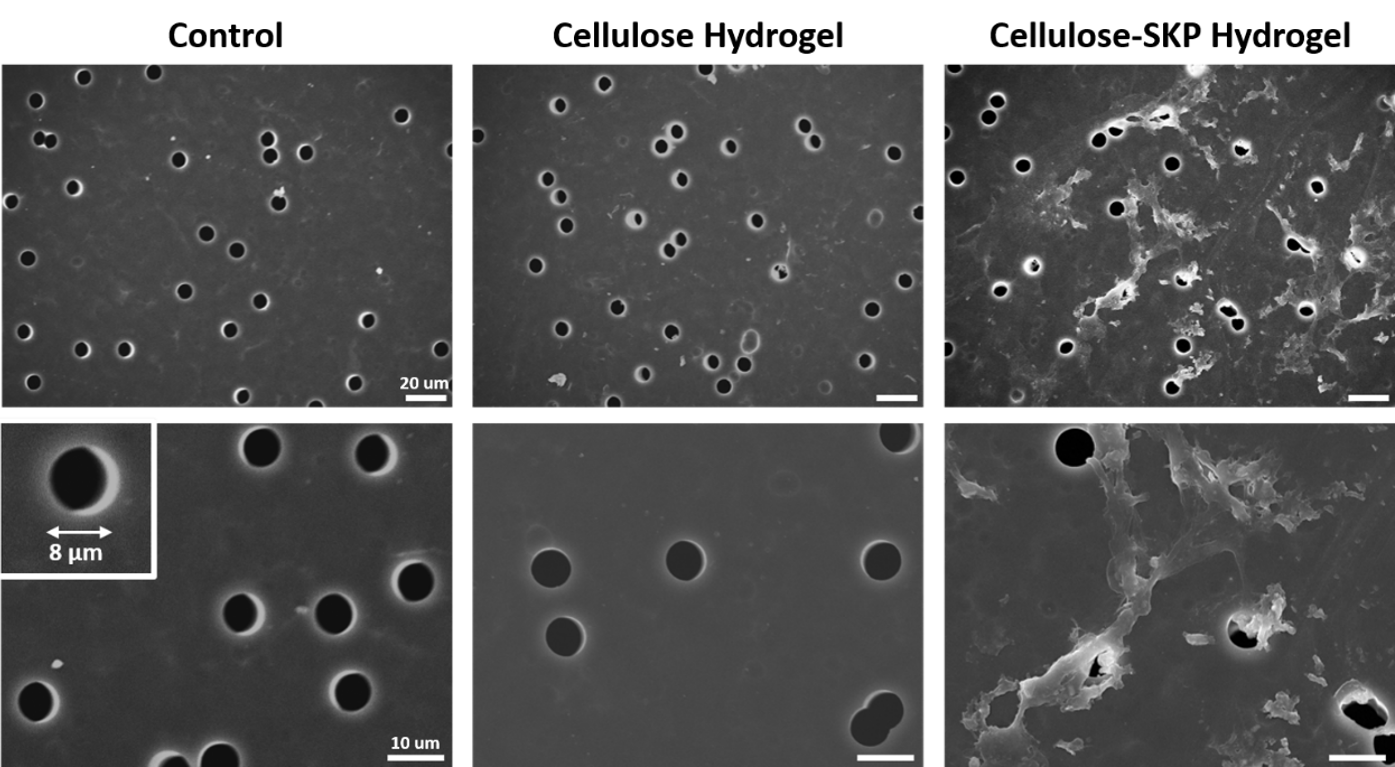


Figure S6. SEM observation of MSC migration at 24 hours. Representative images of Transwell membrane under three conditions in different magnification.


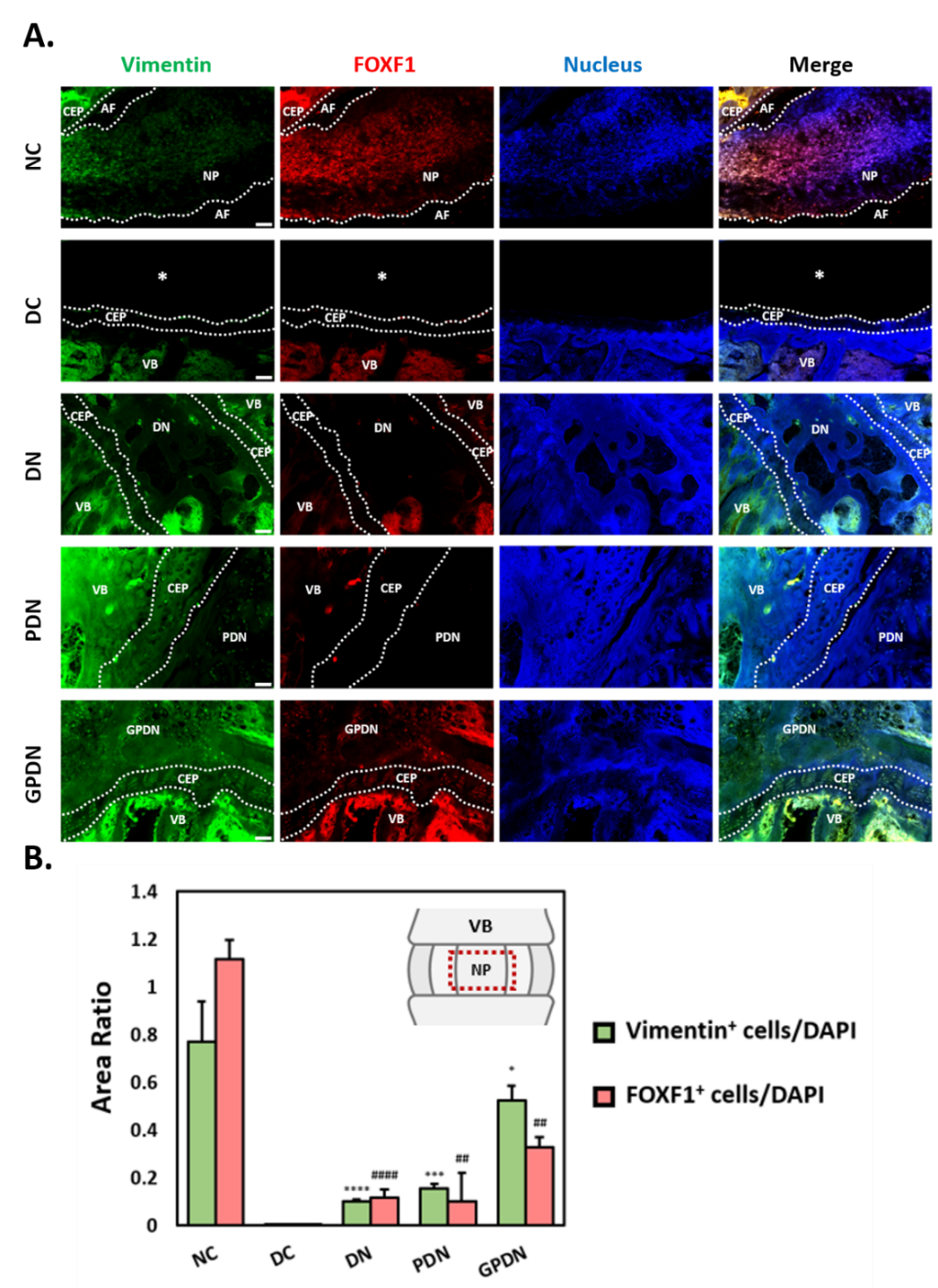


Figure S7. NP-like phenotype differentiation induced by different IVD scaffolds after 4 weeks of implantation. (A) Intervertebral disc and vertebral body sections were stained with Vimentin immunoreactivity (green), FOXF1 NP-like cell staining (red), and merged with DAPI nucleus staining (blue) for different groups. Dotted lines represent boundaries between each part of IVD; star signs represent the empty space caused by native disc discectomy. Scale bars = 50 μm. (VB: vertebral bodies; CEP: cartilage endplate; AF: annulus fibrosus; NP: nucleus pulposus) (B) Vimentin and FOXF1 protein expression was quantified by the area of Vimentin-positive and FOXF1-positive cell signals divided by the area of DAPI signals. Red square outlines the measuring area. Error bars show ± S.D. for total n=3. (* p < 0.05, *** p < 0.001, and **** p < 0.0001 vs negative control in Vimentin group; ## p < 0.01, and #### p < 0.0001 vs negative control in FOXF1 group)


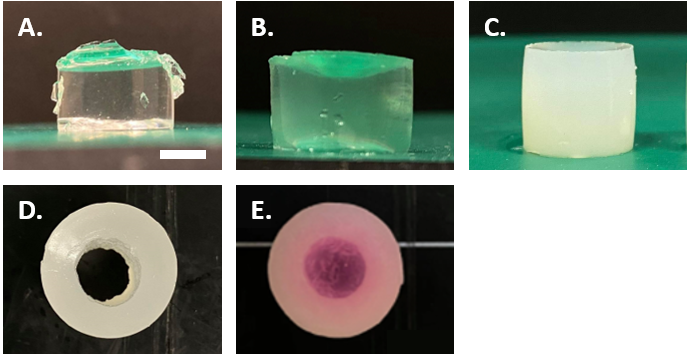


Figure S8. Phenotype of hydrogel samples and IVD scaffolds. (A) Cellulose hydrogel. (B) Cellulose/alginate double network hydrogel (before the immersion of CaCl_2_ solution). (C) Cellulose/alginate double network hydrogel (after the immersion of CaCl_2_ solution). Scale bars = 5 mm.


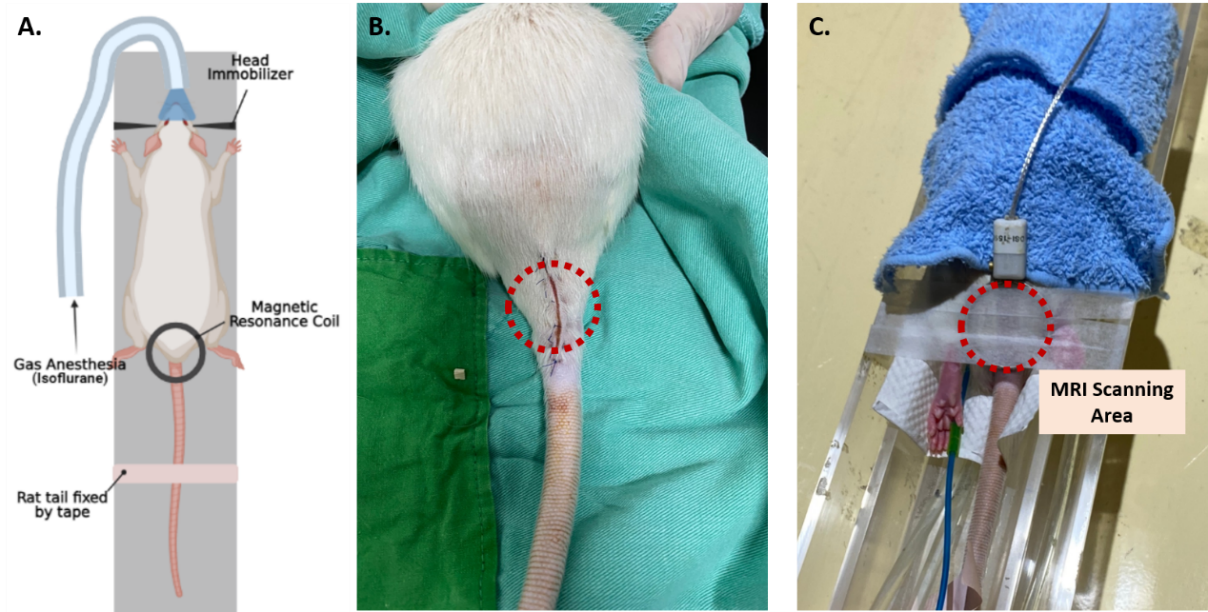


Figure S9. Magnetic resonance imaging (MRI) of rat caudal disc. (A) Schematic representation of experimental animal and MRI carrier. (B) MRI scanning position on rat tail. Red circle shows the implanted scaffold position. (C) Phenotype of MRI operation. Red circle shows the scanning area of MRI.

Table S1. Primers used in quantitative RT-PCR.

| Gene | Forward Primer (5’ to 3’) | Reverse Primer (5’ to 3’) |
| --- | --- | --- |
| *COL2* | CACCGCTAACGTCCAGATGAC | GGAAGGCGTGAGGTCTTCTG |
| *Aggrecan* | GGAATCCCTAGCTGCTTAGCAG | GAGTCATTGGAGCGAAGGTTC |
| *Sox9* | AGGAAGCTGGCAGACCAGTA | ACGAAGGGTCTCTTCTCGCT |
| *KRT19* | GCGAGCTAGAGGTGAAGATC | CGGAAGTCATCTGCAGCCA |
| *GAPDH* | AACGACCCCTTCATTGACCTC | CCTTGACTGTGCCGTTGAACT |

Table S2. Description of each animal experimental group.

| Group | Descriptions |
| --- | --- |
| Negative Conctrol (NC) | Healthy IVD without surgery. |
| Discectomy Control Group (DC) | Native IVD was dissected with saline injection. |
| Double Network Hydrogel Group (DN) | Replaced native IVD with DN implant. (IVD scaffold contains only hydrogel materials) |
| Peptide-functionalized Double Network Hydrogel Group (PDN) | Replaced native IVD with PDN implant. (IVD scaffold contains functional peptides) |
| GDF-5 incorporated, Peptide-functionalized Double Network Hydrogel Group (GPDN) | Replaced native IVD with GPDN implant. (IVD scaffold contains functional peptides and bioactive molecules) |

Table S3. Modified Pfirrmann grading for lumbosacral disc degeneration. Scores ranging from a normal disc with 3 points (1 point in each category) to a severely degenerated disc with 15 points (5 points in each category). The three categories are structure, signal intensity, and the height of intervertebral disc.

| **Grade** | **I. Structure** | **II. Signal Intensity** | **III. Height of IVD** |
| --- | --- | --- | --- |
| 1 | Homogenous, bright white | Hyperintense, isointense to cerebrospinal fluid | Normal |
| 2 | Inhomogenous with or without horizontial bands | Hyperintense, isointense to cerebrospinal fluid | Normal |
| 3 | Inhomogenous, grey | Intermediate | Normal to slightly decreased |
| 4 | Inhomogenous, grey to black | Intermediate to hypointense | Normal to moderately decreased |
| 5 | Inhomogenous, black | Hypointense | Collapsed disc space |

Table S4. Histological grading scale based on four categories of degenerative changes. Scores ranging from a normal disc with 4 points (1 point in each category) to a severely degenerated disc with 12 points (3 points in each category). The four categories are morphology of annulus fibrosus, matrix and morphology of nucleus pulposus, cellularity of nucleus pulposus, and the border between annulus fibrosus and nucleus pulposus.

| I. Morphology of Annular Fibrosus | |
| --- | --- |
| 1 | Well-organized collagen lamellae with no ruptures |
| 2 | Ruptured or serpentine patterned fibers in less than 30% of the anulus |
| 3 | Ruptured or serpentine patterned fibers in more than 30% of the anulus |
| II. Matrix and Morphology of Nucleus Pulposus | |
| 1 | Normal gelatinous appearance; round shape and the NP constitutes more than 50% of the disc area |
| 2 | Slight condensation of the extracellular matrix; NP constitutes 50% to 25% of the disc area |
| 3 | Moderate/severe condensation of the extracellular matrix; irregular shape and the NP constitutes less than 25% of the disc area |
| III. Cellularity of Nucleus Pulposus | |
| 1 | Normal cellularity with large vacuoles in the gelatinous structure of the matrix, stellar-shaped cells with a proteoglycan matrix located at the periphery, evenly distributed |
| 2 | Slight decrease in the number of cells and fewer vacuoles |
| 3 | Moderate/severe decrease (50%) in the number of cells and no vacuoles, mostly large, round cells, separated by dense areas of proteoglycan matrix |
| IV. Border between the Anulus Fibrosus and Nucleus Pulposus | |
| 1 | Normal |
| 2 | Minimally interrupted |
| 3 | Moderate/severe interruption |
